# *massMatchR*: a Shiny application for glycomics mass spectrometry data analysis

**DOI:** 10.1093/bioinformatics/btag700

**Published:** 2026-09-22

**Authors:** Gábor Beke, Lubos Klucar, Zuzana Pakanová, Rebeka Kodríkova, Peter Baráth, Marek Nemčovič

**Affiliations:** Institute of Chemistry, Slovak Academy of Sciences, 845 38 Bratislava, Slovakia; Institute of Molecular Biology, Slovak Academy of Sciences, 845 51 Bratislava, Slovakia; Institute of Molecular Biology, Slovak Academy of Sciences, 845 51 Bratislava, Slovakia; Institute of Chemistry, Slovak Academy of Sciences, 845 38 Bratislava, Slovakia; Institute of Chemistry, Slovak Academy of Sciences, 845 38 Bratislava, Slovakia; Institute of Chemistry, Slovak Academy of Sciences, 845 38 Bratislava, Slovakia; Institute of Molecular Biology, Slovak Academy of Sciences, 845 51 Bratislava, Slovakia

## Abstract

**Motivation:**

The identification and analysis of glycans using MALDI-TOF mass spectrometry is a critical task in glycomics research, yet it often requires complex data interpretation and manual processing. Existing software tools frequently lack automated solutions for efficient glycan annotation, data structuring and grouping, and semi-quantitative evaluation, making large-scale glycan analysis challenging. To address these limitations, we developed *massMatchR*, an open-source software tool designed to streamline the identification and quantification of glycans in MALDI-TOF spectra.

**Results:**

*massMatchR* automates glycan identification by mapping experimentally detected *m/z* values from preprocessed MALDI-TOF-MS datasets to glycans from a user-defined database. The software provides both visual and tabular outputs, enabling rapid and accurate glycan interpretation. Additionally, *massMatchR* facilitates the export of structured data tables (e.g. Microsoft Excel) with predefined fields for *m/z* and intensity, supporting further analysis. A key feature of the software is its ability to perform semi-quantitative evaluation based on relative intensity calculations, allowing for comparisons across multiple samples. Freely available at http://www.imb.savba.sk/soft/massMatchR/ and on GitHub implemented in R, *massMatchR* offers a fast, reproducible, and customizable computational framework for glycomics and MALDI-TOF-based glycan analysis.

**Availability and implementation:**

An implementation code is available on Github at https://github.com/bekegbor/massMatchR and on Zenodo at https://doi.org/10.5281/zenodo.20697072.

## 1 Introduction

Mass spectrometry (MS) is a cornerstone of modern glycomics, but the structural complexity of glycans, their diversity, and derivatization-based modifications pose significant challenges for automated data interpretation. To overcome these challenges, various computational tools have been developed to support glycan identification, spectral annotation, and quantitative evaluation—each employing different algorithmic strategies to enhance accuracy and efficiency.

Several software solutions have been introduced to support different stages of glycomic mass spectrometry workflows, ranging from spectral preprocessing and quality control to glycan annotation and comparative analysis. For MALDI-TOF-MS preprocessing, commercial software such as flexAnalysis software v3.4 (Bruker Daltonics, Bremen, Germany) is commonly used for spectral visualization, peak picking, and initial data processing. In parallel, the open-source software MassyTools provides automated spectrum calibration, data extraction, and quality control for high-throughput glycomic and glycoproteomic datasets (Jansen *et al.* 2015).

For glycan assignment, AssignMALDI is designed specifically for MALDI-TOF MS data, allowing users to create glycan databases that incorporate customized derivatization strategies and automatically assign glycan masses based on these modifications (Mehta *et al.* 2020). For high-throughput glycan analysis using LC-MS/MS, GlyKAn AZ was developed as a MATLAB-based application that integrates MS1 and MS2 mass search algorithms to confirm glycan structures with high accuracy (Singh *et al.* 2021). Other tools such as GlycoWorkbench focus on semi-automated annotation, where users propose candidate glycan structures that are then evaluated against observed masses or annotate peaks with structures from various databases (Ceroni *et al.* 2008). However, this tool does not directly support visualization and comparison across multiple samples or experimental groups. GRITS Toolbox was developed to handle large glycomics datasets by integrating automated glycan annotation with metadata storage and post-processing capabilities (Kronewitter *et al.* 2018). This tool enables side-by-side comparisons of different MS runs, which is essential for comparative glycomics studies, though it still relies on predefined glycan databases that may limit its applicability to novel glycoforms. Such computational approaches are particularly valuable in clinical glycomics, for instance in the study of congenital disorders of glycosylation (CDG), where detailed and comparative analysis of *N-* and *O-glycan* profiles is essential to understand disease mechanisms and identify potential biomarkers.

Despite the advancements made by these tools, there remains a need for software that combines high-speed spectral annotation, automated database matching, quantitative evaluation, and comparison between individual samples or groups, while also being accessible, reproducible, customizable, and at the same time accessible to non-programmers. To address these challenges, we introduce *massMatchR*, an open-source Shiny/R-based downstream post-processing tool developed for automated identification of glycans in MALDI-TOF MS spectra. It employs user-defined glycan databases and preprocessed spectral data with assigned peaks to facilitate rapid mass-to-glycan matching. It provides results both in tabular form and as interactive bar charts for data interpretation. Unlike existing solutions, *massMatchR* integrates semi-quantitative analysis based on relative intensity calculations, allowing for large-scale comparisons across multiple samples. Unlike preprocessing-oriented tools such as MassyTools or visualization-focused environments such as GlycoWorkbench, *massMatchR* was designed as a lightweight downstream post-processing platform that integrates automated glycan matching, structured data export, semi-quantitative relative intensity evaluation, and comparison across multiple samples within a reproducible workflow. Freely available on GitHub, *massMatchR* is a lightweight, customizable tool for streamlined glycomics analysis, offering enhanced speed, automation, and reproducibility in MALDI-TOF MS-based research.

## 2 Methods

*massMatchR* was developed in R, version 4.4.1 on Microsoft Windows 10 (R Core Team 2021) using the shiny package (Chang *et al.* 2026) and other packages: shinyBS (Bailey 2022), shinyjs (Attali 2021), htmlwidgets (Vaidyanathan *et al.* 2023), openxlsx (Schauberger and Walker 2023), DT (Xie *et al.* 2025), tableHTML (Boutaris *et al.* 2023), ggplot2 (Wickham 2016), plotly (Sievert 2020), and colourpicker (Attali 2023). These packages provide the interactive frontend, file handling, and visualization features required for dynamic analysis. The *massMatchR* overall workflow, illustrating the inputs, processing steps, and outputs, is shown in Fig. 1.

**Figure 1:**
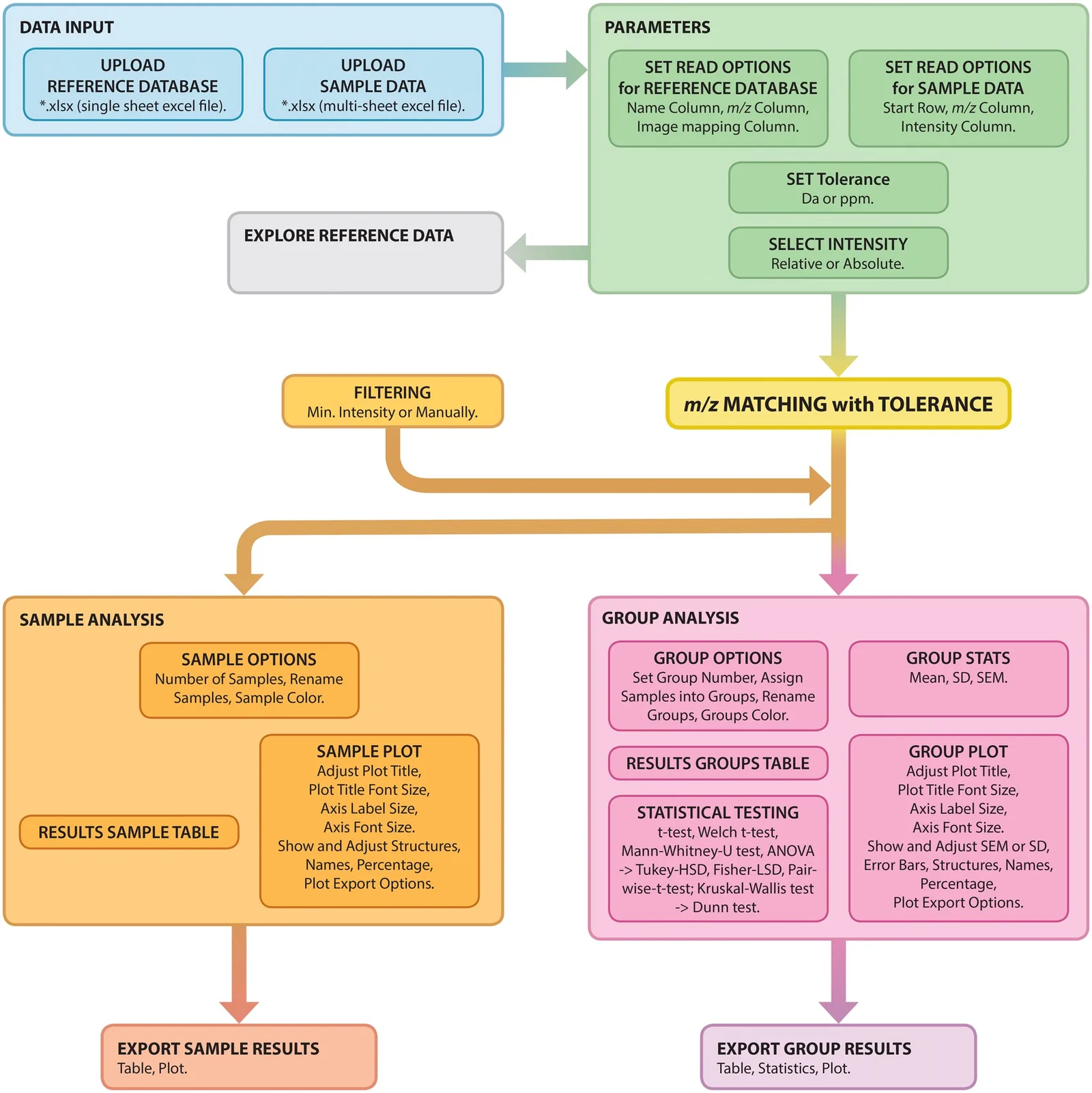
*massMatchR* Workflow scheme. The scheme illustrates the overall *massMatchR* pipeline.

*massMatchR* is available online at http://www.imb.savba.sk/soft/massMatchR/. Also, the source code is publicly available on GitHub (https://github.com/bekegbor/massMatchR) and on Zenodo (https://doi.org/10.5281/zenodo.20697072), along with installation instructions, documentation, and example datasets. A comprehensive step-by-step user manual describing data upload, parameter settings, mass matching, filtering, visualization, grouped sample analysis, statistical testing, and result export is provided as Supplementary File 1, available as supplementary data at *Bioinformatics* online and is also available through the GitHub repository. Online version of *massMatchR* is defaulted to a set of 331 theoretical *N-*glycans based on the human serum *N-*glycome library (Kronewitter *et al.* 2009), which represents a reliable basis for accurate glycan profiling. The downloadable GitHub version is fully user-accessible, enabling the integration of custom *N-*glycan databases for flexible data analysis. Furthermore, both graphical illustration and symbol nomenclature are fully aligned with Consortium for Functional Glycomics (CFG) guidelines with the aim to cope better with the rapidly growing information on the structure and functions of glycans in chemical and biological systems (Varki *et al.* 2015). Graphical representations of selected glycans are stored on the server and function fully in the online version. These *N-*glycan structures were generated in GlycoWorkbench and should be considered strictly theoretical, representing the most probable structures. Glycan assignments were based on compositions reported by Kronewitter *et al.*, with the theoretical match chosen for visualization only.

When using the GitHub version, users have the option to edit the images or upload custom images to adapt the visualization to their specific needs. Reference data can be uploaded as a single-sheet Microsoft Excel file containing at least one column with *m/z* values, which serve as the basis for matching experimental data. Additional columns, such as Name or supplementary *m/z* entries, may be included to link glycan structures to their respective masses. This format accommodates multiple mass modifications of a single glycan (e.g. permethylation), while structural assignments are consistently based on the raw *m/z* values. Importantly, the application remains fully functional even when glycan names or structural annotations are not present in the reference database. In such cases, mass matching and downstream quantitative analyses are performed solely based on *m/z* values. This is particularly relevant for theoretical glycan libraries, such as the Kronewitter dataset (Kronewitter *et al.* 2009), which does not include explicit textual or graphical structural annotations.

Experimental data are uploaded as a multi-sheet Microsoft Excel file, where each sheet represents an individual sample. Such input tables may originate from external preprocessing software, including flexAnalysis, MassyTools, or other MALDI-TOF MS data extraction pipelines. The only requirement is that all sheets share the same column structure (e.g. number, order, and header names). *massMatchR* matches each experimental mass to entries in the reference database within a user-adjustable tolerance in Da or parts per million (ppm). This tolerance, like all other key parameters, can be adjusted in real time, and the interface instantly updates the results to reflect any changes, which is a feature not commonly available in other glycan data analysis software. Following mass matching, *massMatchR* computes the relative intensity for each identified glycan within each sample.

To reduce the need for input table preprocessing, users can flexibly select which columns to use for the matching step. This feature is particularly useful when input files exported from other software include columns, which would not be used in *massMatchR* (e.g. signal-to-noise ratio, quality factor, and other signal parameters).

A key limitation in glycan annotation based solely on accurate mass is the presence of isobaric glycan structures, which are a well-known challenge in glycomics. Furthermore, distinct compositions such as di-fucosylated and monosialylated species can exhibit similar masses, making them difficult to distinguish at the MS1 level. In *massMatchR*, this limitation is addressed by reporting a single best-matching glycan per observed *m/z* value, selected based on the closest theoretical mass within a defined tolerance. However, the discrimination of isobaric structures is not possible using mass alone and would require an additional approach such as MS/MS fragmentation, exoglycosidase digestion, or chromatographic separation. The software relies on guided annotation using a user-defined, curated glycan library that reflects the expected biological glycome, where each glycan is assumed to correspond to a unique mass entry. Although MS/MS-based annotation could improve structural differentiation, *massMatchR* does not use fragmentation data and instead focuses on fast precursor *m/z*-based matching against curated libraries, enabling rapid selection of relevant glycan signals for the analysis of relative intensity data and removal of contaminant ions.

### 2.1 Grouped sample analysis and variability estimation

After matching glycan peaks across all individual samples, *massMatchR* allows users to group samples for comparative analysis. This is useful in studies comparing experimental cohorts, such as patient versus control groups or different treatment conditions. Grouping is done through a simple checkbox interface, where each sample can be assigned to a user-defined group. Once grouped, the application calculates the average intensity of each glycan across all samples in that group. Groups can be edited or redefined at any time without needing to reload the data, and all outputs—tables and plots—update instantly.

To assess variability within groups, users can choose between two statistical measures: standard deviation (SD) or standard error of the mean. These values are automatically calculated and displayed in both the results table and as error bars on group-level bar charts, helping users visually assess the consistency of glycan expression within each group.

To support comparative glycomics analysis, *massMatchR* incorporates a built-in statistical testing module that automatically adapts the available tests based on the number of defined groups and the assumed data distribution. For two-group comparisons under parametric assumptions, users may choose between an independent samples t-test (equal variances) or a Welch t-test (unequal variances), while the non-parametric alternative available is the Mann–Whitney U (Wilcoxon rank-sum) test. When three or more groups are compared, one-way analysis of variance is offered for parametric data, followed by post-hoc test options including Tukey HSD, Fisher LSD, and pairwise t-tests with either Bonferroni or Benjamini–Hochberg (BH/FDR) correction. For non-parametric multi-group comparisons, the Kruskal–Wallis test is applied, followed by Dunn’s post-hoc test from the dunn.test package (Dinno 2026). For each glycan, the module reports *P*-values, adjusted *P*-values (either with Bonferroni or Benjamini–Hochberg correction), log2 fold-change values calculated with the gtools package (Bolker *et al.* 2022) for each pairwise comparison, and significance flags based on user-defined thresholds for *P*-value and |log2FC| (the default values are *P* < 0.05 and |log2FC| ≥ 1). The results of the statistical analysis are presented in a downloadable table, enabling researchers to rapidly identify glycans with statistically and biologically meaningful differences between groups.

### 2.2 User interface design

The application provides an interactive and responsive user experience, dynamically updating all outputs in response to modifications in inputs or filtering conditions. The *massMatchR* user interface is divided into three main panels:

*Data input and parameter selection—*This section includes controls for uploading reference and sample data, selecting matching columns, setting mass tolerance, and adjusting other core parameters.
*Data filtering*—To refine the matched results, *massMatchR* offers intensity-based filtering, where glycans falling below a user-defined relative intensity threshold are excluded from both the results table and visualizations, either with or without recalculation of relative intensities across the remaining matched peaks. Additionally, filtering can be applied to suppress the display of glycan structure images, names, and percentage annotations above the bars in the interactive bar chart, without altering the underlying dataset.
*Results visualization*—Results are presented across two tabs—Samples and Groups. The Samples tab displays matched results for each sample individually, while the Groups tab enables users to group samples and compare averaged intensities across groups. Both views include downloadable tables and interactive bar charts (Fig. 2). Additional controls allow customization of plot appearance (e.g. colors, titles, font sizes) and sample metadata (e.g. renaming).

**Figure 2:**
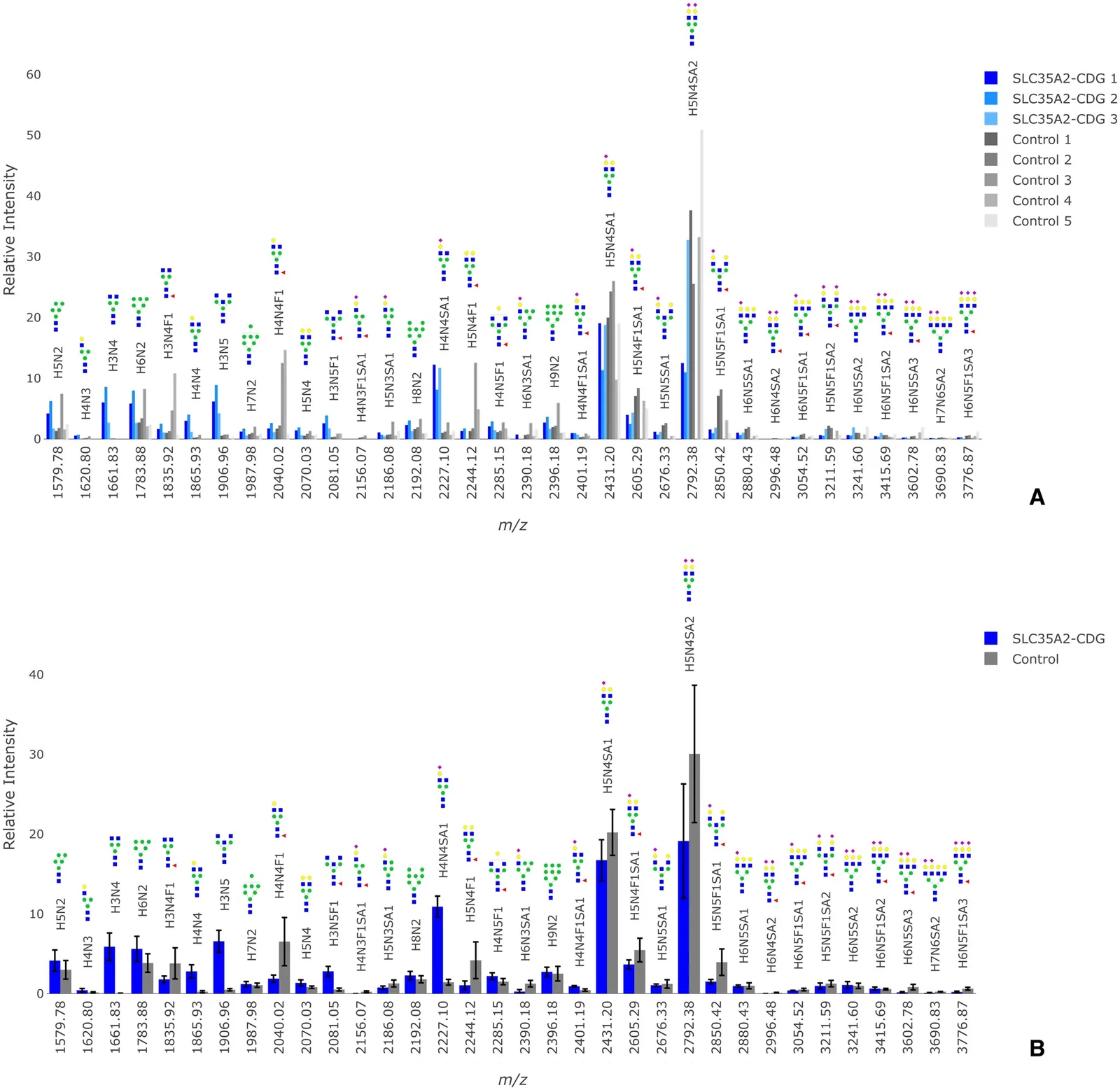
Visualization of testing data (case study—comparison of relative intensities of permethylated serum *N*-glycoprofiles of SLC35A2-CDG patient and control samples) presented as bar graphs using *massMatchR*. (A) Individual samples from the control (*n* = 3) and patient (*n* = 5). (B) Samples grouped and displayed by category: Control and patient, both shown with standard deviation. F—fucose; N—*N*-acetylhexosamine; H—hexose; SA—*N*-acetylneuraminic acid. *Note*: These N-glycan structures were generated in GlycoWorkbench and should be considered strictly theoretical, representing the most probable structures.

## 3 Results

### 3.1 Case study

To evaluate the performance of the presented computational application, previously published data (Kodríková *et al.* 2023) were used as input. The dataset included raw MALDI-TOF data from three replicates of permethylated serum *N*-glycans from a patient with SLC35A2-CDG and from five controls, enabling assessment of differences in the patient’s *N*-glycoprofile. Manually conducted reference database of the 80 most frequently observed *N*-glycan species in human serum, covering major oligomannosidic, hybrid, and complex *N*-glycan types, including textual labels, was used as input. Example data from this case study are available at https://github.com/bekegbor/massMatchR.

Each sample was uploaded as a separate sheet in a multi-tab Excel file exported from FlexAnalysis software program (Bruker, DE) with *m/z* and intensity columns specified for matching. With a specified mass tolerance of 0.5 Da, an average of 60 glycans per sample was identified by matching the experimental data with the reference glycan table. Bar plots revealed changes in several *N*-glycan structures, including galactosylated and sialylated forms. To better visualize these changes, samples were grouped, relative intensities were averaged, and SDs were calculated. *massMatchR* detected significant group differences, showing a marked increase in hypogalactosylated glycan structures in SLC35A2-CDG samples, including H3N4, H4N4, and H4N4SA1, as well as two additional agalactosylated glycans, H3N5 and H3N5F1, compared to control samples. To validate the statistical module, an independent samples t-test was performed on the case study data (SLC35A2-CDG group vs. control group), matched against the Kronewitter *et al.* (2009) reference database, which contains approximately 2.5 times as many theoretical masses as the in-house database used in the original case study. Despite the difference in reference data, the same glycans were identified as statistically significant, with the larger reference database additionally yielding a higher number of significant results due to the increased number of matched structures. Thus, *massMatchR-*based data analysis confirmed the findings reported in the original study, with the glycan profiling results clearly supporting a deficiency in the galactose incorporation in SLC35A2-CDG patient. In addition to the whole-serum glycomics data presented here, the general workflow can also be extended to targeted glycomic analyses, such as immunoglobulin-derived *N-*glycomes, through the implementation of customized glycan databases. This case study demonstrates how *massMatchR* can streamline hypothesis-driven glycomics analysis and provide interpretable outputs suitable for both research and clinical workflows.

### 3.2 Generalization and reusability

Although *massMatchR* was originally developed to identify and semi-quantify glycans from MALDI-TOF mass spectrometry data, its core functionality goes beyond glycomics. *massMatchR* compares two or more sets of structured data—user-uploaded reference table and one or more sample tables—based on selected columns and a numerical tolerance. This makes it useful for many other types of data matching, peak identification, or data alignment tasks. *massMatchR* can be adapted to work with other types of molecules, such as lipids, metabolites, peptides, or nucleotides, if a reference table (database) is available. It can even be used outside of mass spectrometry, e.g. clinical biomarker data matching. As long as the data are organized in tables with numeric and/or text columns, *massMatchR* can help users quickly match, annotate, and compare values across datasets. Thanks to this flexibility, it can be used in a wide range of scientific and analytical fields.

## Supplementary Material

btag700_Supplementary_Data

## Data Availability

The source code, documentation, and example datasets underlying this article are publicly available in the *massMatchR* GitHub repository at https://github.com/bekegbor/massMatchR and are archived on Zenodo at https://doi.org/10.5281/zenodo.20697072.
