# Correction: *massMatchR*: a Shiny application for glycomics mass spectrometry data analysis

**DOI:** 10.1093/bioinformatics/btag770

**Published:** 2026-10-19

This is a correction to: Gábor Beke, Lubos Klucar, Zuzana Pakanová, Rebeka Kodríkova, Peter Baráth, Marek Nemčovič, *massMatchR*: a Shiny application for glycomics mass spectrometry data analysis, *Bioinformatics*, Volume 42, Issue 10, October 2026, btag700, https://doi.org/10.1093/bioinformatics/btag700

The following change has been made to the originally published paper. The names of the second co-author have been corrected to read: “Lubos Klucar”.

