# Supplementary material for: massMatchR: a Shiny application for glycomics mass spectrometry data analysis
Source: Bioinformatics. 2026 Sep 22;42(10):btag700. doi: 10.1093/bioinformatics/btag700 (PMC13637607; doi:10.1093/bioinformatics/btag700)
Supplement: Supplementary file 1 [file btag700_supplementary_data.pdf]

massMatchR User Manual

massMatchR is an interactive R Shiny application for matching mass spectrometry (MS) peak lists against a user-defined reference *m/z* database within a specified tolerance window. *massMatchR* is available online at http://www.imb.savba.sk/soft/massMatchR/[1]. The source code and release archive are also available from GitHub[2] or Zenodo[3], respectively, allowing the application to be installed and run locally. For local installation instructions, see the Local Installation Guide on GitHub[4].

Although the hosted examples and structure-image features are glycan-focused, the core matching workflow can also be applied to other reference datasets that use numerical tolerance matching.

This manual explains how to prepare the reference and sample workbooks, configure the matching parameters, inspect the reference data and matched results, and export tables and plots.

Table of Contents

1 How massMatchR works ... 2
2 Setting up the analysis ... 3
- 2.1 Interface overview ... 3
- 2.1.2 Main Panel ... 6
- 2.2 Upload and configure the reference database ... 7
- 2.3 Explore reference data ... 9
- 2.4 Upload and configure the sample data ... 10
- 2.5 Matching ... 12

3 Sample analysis ... 14
- 3.1 Selecting the number of samples ... 14
- 3.2 Renaming samples ... 15
- 3.3 Setting sample colors ... 16
- 3.4 Sample results table ... 17
- 3.5 Filtering the matched results ... 18
- 3.6 Sample plot ... 20

4 Group analysis ... 26
- 4.1 Creating groups and assigning samples ... 26
- 4.2 Group results table ... 27
- 4.3 Group plot and error bars ... 29
- 4.4 Group statistical analysis ... 31

[1]http://www.imb.savba.sk/soft/massMatchR/
[2]https://github.com/bekegbor/massMatchR
[3]https://zenodo.org/records/20697073
[4]https://github.com/bekegbor/massMatchR/blob/main/docs/LOCAL_INSTALL.md

1 How *massMatchR* works

massMatchR performs tolerance-based nearest-peak matching in three stages:

1. The application reads the selected reference *m/z* column. Rows with missing *m/z* values are excluded. For matching, the *m/z* values are rounded to four decimal places, and duplicate rounded values are collapsed by retaining the first occurrence.
2. For each reference *m/z* value, *massMatchR* searches each selected sample worksheet for measured peaks within the selected tolerance window. If multiple peaks fall within the window, the peak with the smallest *m/z* difference is selected and its intensity is recorded. If no peak is found within the tolerance range, the intensity is set to 0 (no match).
3. Reference *m/z* values that are not detected in any sample are removed from the results.

The complete *massMatchR* workflow is summarized in Figure 1.

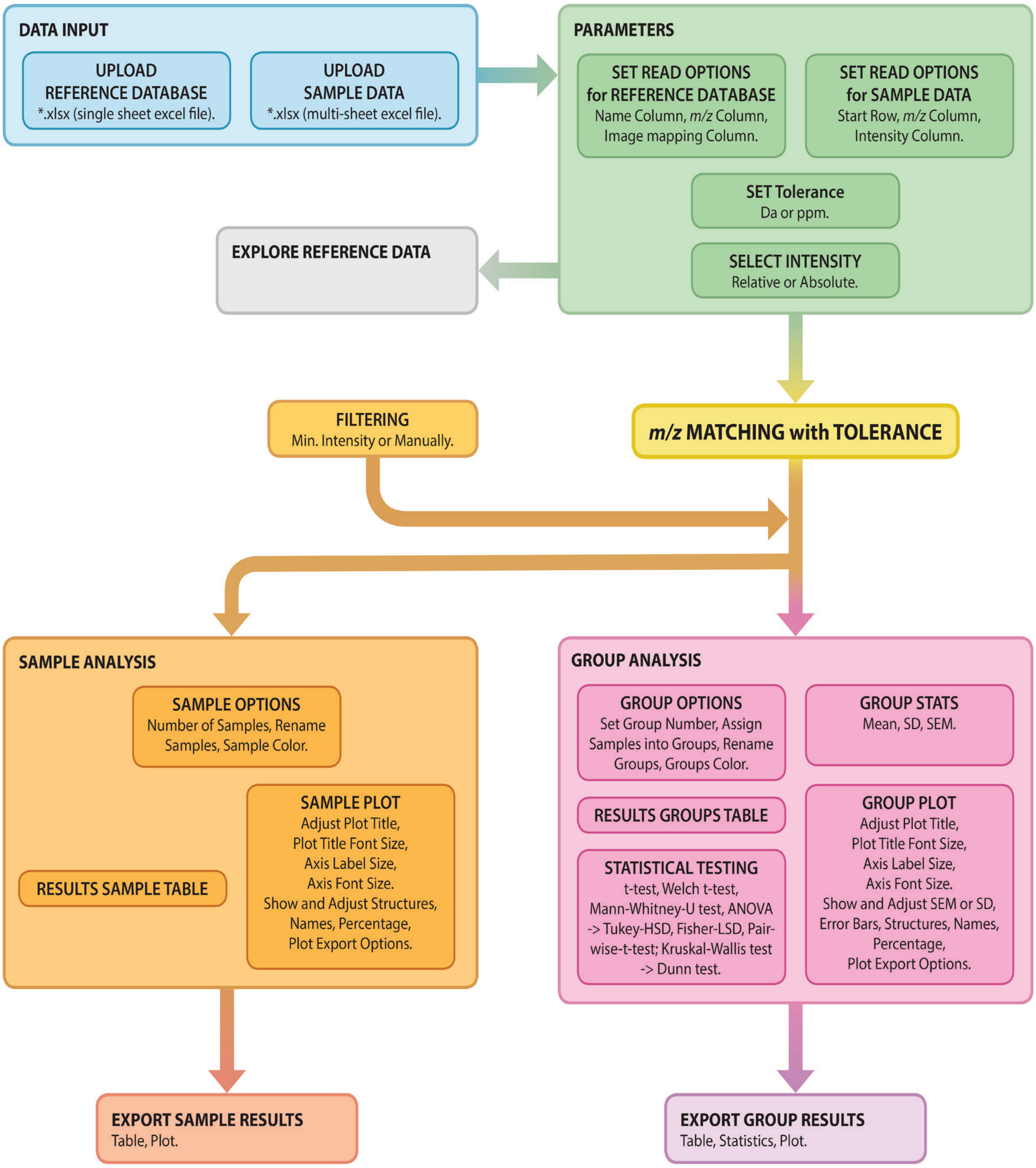

Figure 1. Overview of the *massMatchR* workflow.

2 Setting up the analysis

2.1 Interface overview

To follow the examples in this manual, download the files from the example_data[5] directory of the *massMatchR* GitHub repository.

When the application opens, the main interface is divided into two areas (Figure 2):

- Sidebar Panel (Figure 2A), which contains the input-file and matching controls.
- Main Panel (Figure 2B), which contains the analysis, visualization, and reference-data tabs.

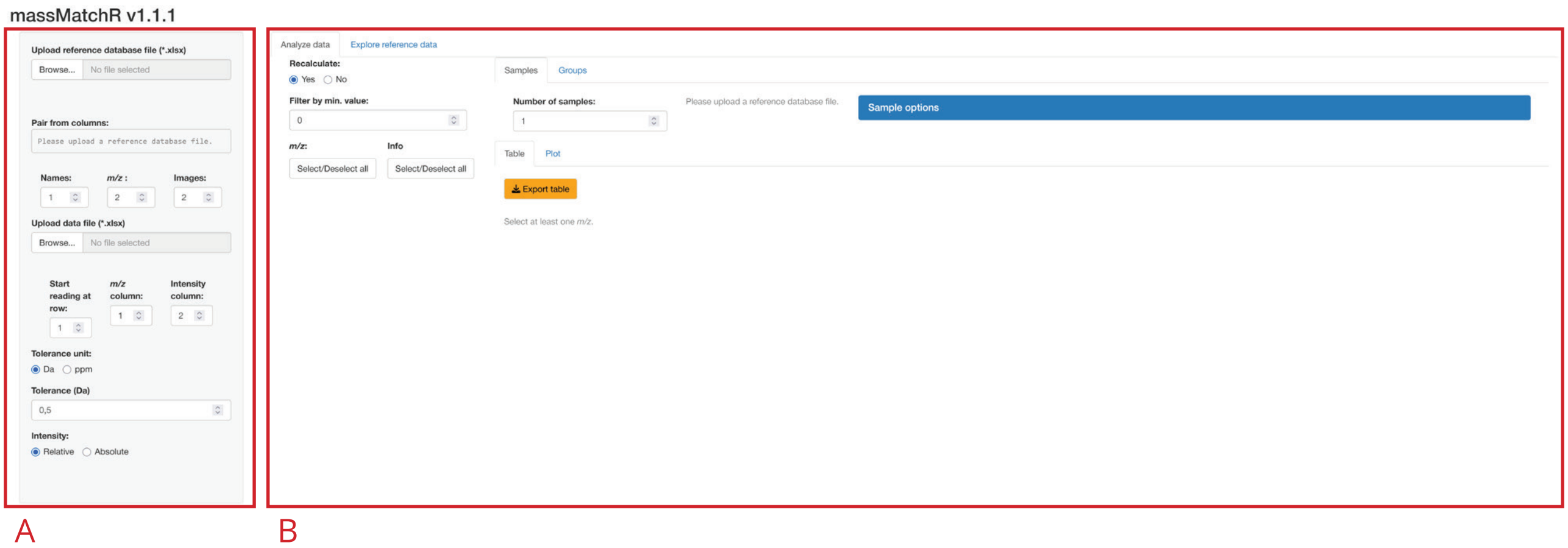

Figure 2. Main interface of the *massMatchR* application: (A) sidebar panel; (B) main panel.

[5]https://github.com/bekegbor/massMatchR/tree/main/example_data

Display notice

The *massMatchR* interface was developed and tested on a 16:9 display with a resolution of 2560 × 1440 pixels. On screens with a lower resolution, in a small browser window, or when the browser zoom is set above 100%, some controls may appear partially clipped or wrap onto additional lines (Figure 3). This is only a display issue and does not alter the underlying calculations.

Before beginning the analysis:

1. Maximize the browser window.
2. Check that the browser zoom is set to 100%. In most browsers, open the browser menu and locate the Zoom setting. If some controls are still clipped, reduce the browser zoom to 90% or 80% until the complete interface is visible. The current zoom percentage may also be displayed in the browser address bar or menu when it differs from 100%. Common keyboard shortcuts are:
   - Windows and Linux: Press Ctrl + 0 to reset the zoom to 100%. Use Ctrl + - to zoom out and Ctrl + + to zoom in.
   - macOS: Press Command + 0 to reset the zoom to 100%. Use Command + - to zoom out and Command + + to zoom in.
3. If necessary, check that the operating system is using the display's recommended resolution.
   - On Windows, open: Settings -> System -> Display. Check the Display resolution and Scale settings. Use the recommended resolution whenever possible. A high display-scaling value may cause the interface to occupy more screen space.
   - On macOS, open: System Settings -> Displays. Select the default resolution or a setting that provides more screen space if parts of the application are not visible.

Reducing the browser zoom is usually the simplest solution and does not require changing the system-wide display settings.

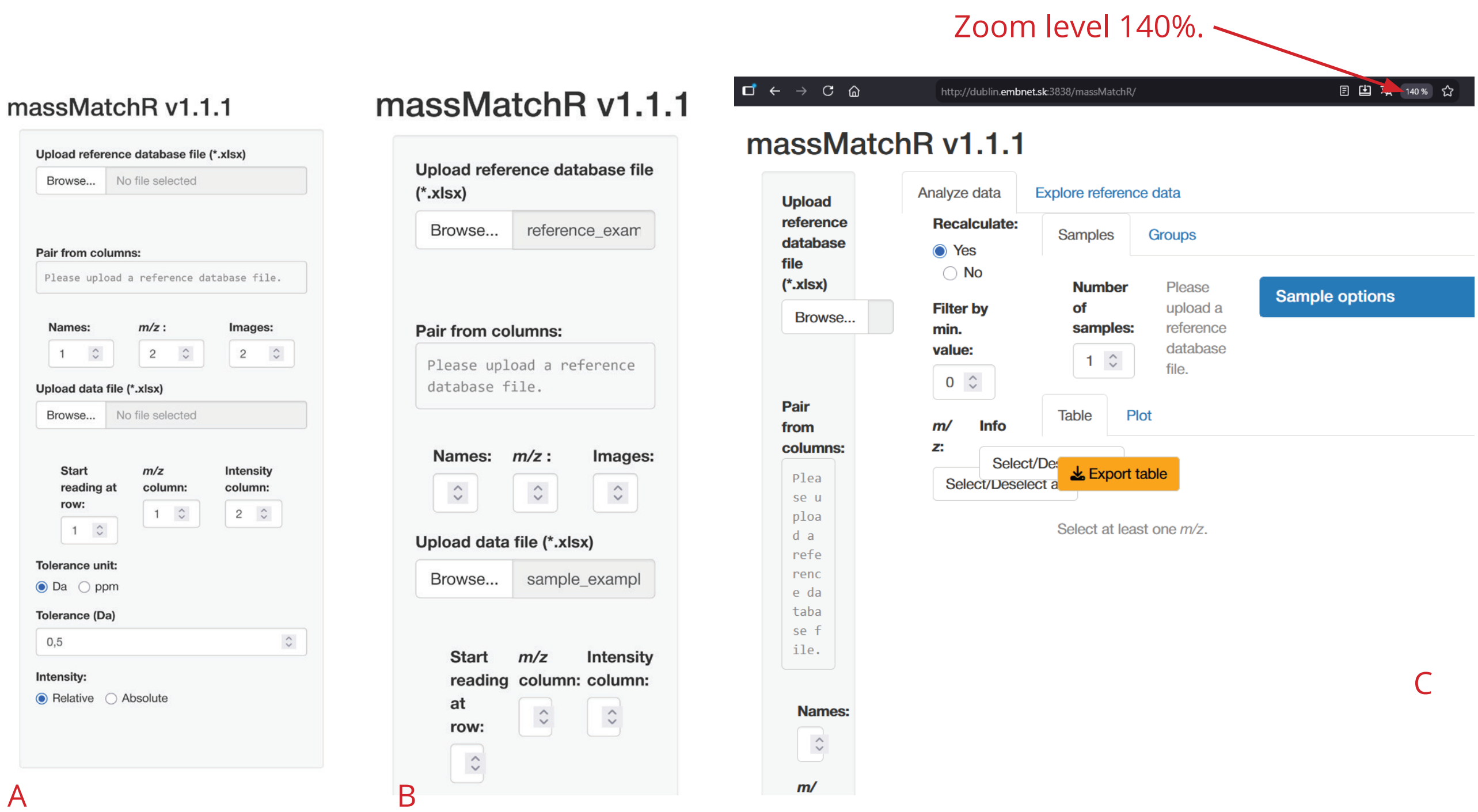

Figure 3. Effect of browser zoom on the *massMatchR* interface: (A) expected layout at 100% zoom; (B) clipped controls at 140% zoom; (C) controls wrapped onto additional lines at 140% zoom.

2.1.1 Sidebar panel

The sidebar panel contains the controls used to load the input workbooks and configure the matching settings (Figure 4):

1. **Upload reference database file:** Uploads the reference workbook in *.xlsx* format.
2. **Pair from columns:** Lists the columns detected in the reference workbook (single sheet) together with their indices.
3. **Names:** Specifies the index of the reference column used to label entries in tables and plots.
4. ***m/z*:** Specifies the index of the theoretical or expected *m/z* column used for matching.
5. **Images:** Specifies the index of the column whose values are used to identify structure images.
6. **Upload data file:** Uploads the multi-worksheet sample workbook in *.xlsx* format.
7. **Start reading at row:** Specifies the row containing the column headers in every sample worksheet. The default is row 1.
8. ***m/z* column:** Specifies the index of the column containing the measured *m/z* values in every sample worksheet.
9. **Intensity column:** Specifies the index of the column containing the measured intensity values.
10. **Tolerance unit:** Selects either daltons (Da) or parts per million (ppm).
11. **Tolerance:** Specifies the maximum permitted difference between a reference value and a measured peak. The default is 0.5 Da or 20 ppm, depending on the selected unit.
12. **Intensity:** Selects whether matched intensities are reported as relative percentages or as absolute measured values.

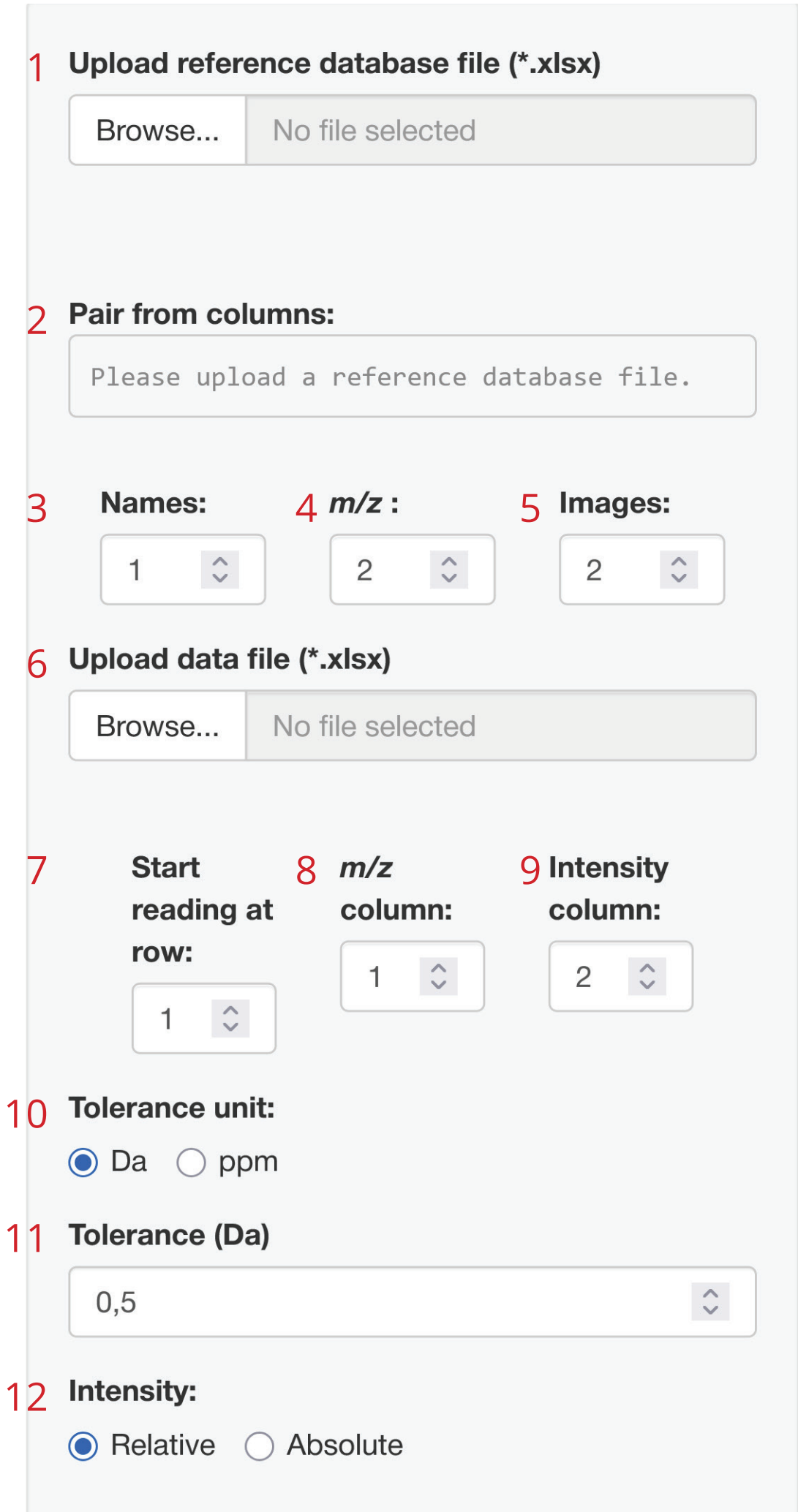

Figure 4. Controls in the sidebar panel. The numbered labels correspond to the list above.

2.1.2 Main Panel

The main panel contains the application's data-analysis and reference-data workspaces. It is divided into two primary tabs:

- Analyze data
- Explore reference data

The Analyze data tab contains the controls and outputs used after the reference and sample workbooks have been uploaded. Its left side contains the shared analysis controls, including:

- Relative-intensity recalculation
- The minimum-value plot filter
- Selection or deselection of matched reference *m/z* values
- Selection or deselection of supplementary information displayed above plot bars

These controls affect the analyses presented in the Samples and Groups tabs. The right side of the Analyze data tab we can find:

- The Samples tab contains the individual-sample results. It provides controls for selecting the number of worksheets to analyze, renaming samples, assigning sample colors, viewing the results table, and creating the sample plot.
- The Groups tab is used to assign samples to groups and calculate group-level results. It contains the Group Table, Group Plot, and Group Statistics tabs.

The Explore reference data tab displays the selected reference names, reference *m/z* values, and available structure images. It can be used to confirm that the reference workbook and column selections were interpreted correctly before continuing with the analysis.

Detailed instructions for the Samples and Groups tabs are provided in the following chapters.

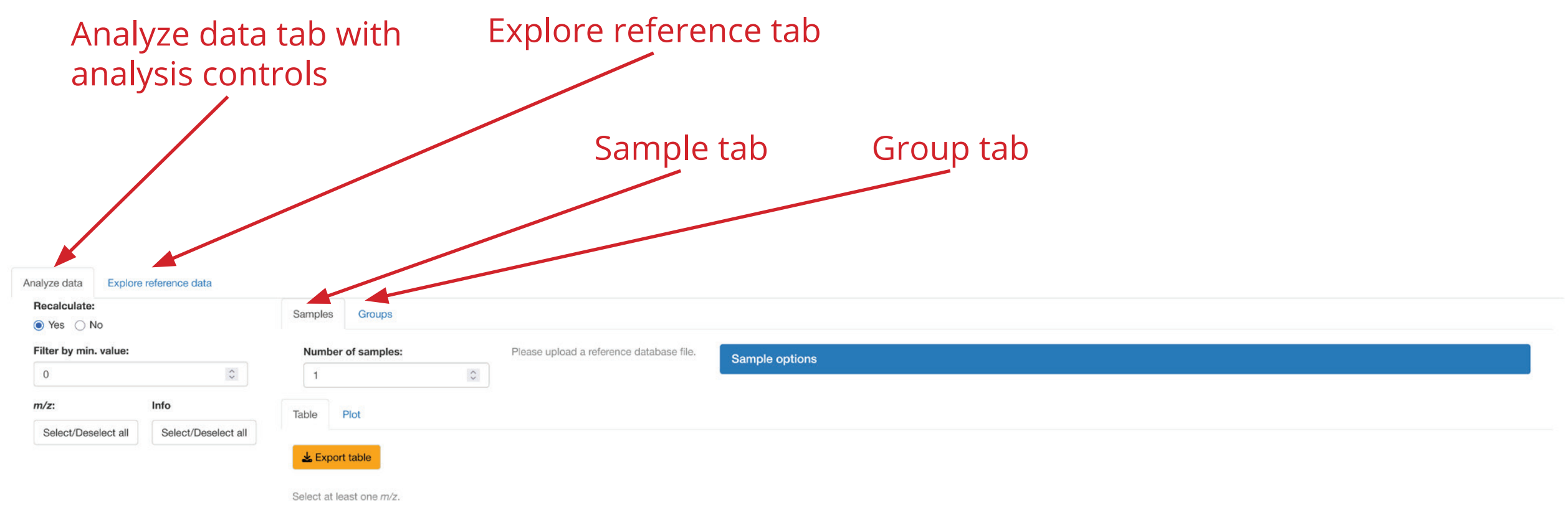

Figure 5. Main panel of the *massMatchR* application, showing the Analyze data and Explore reference data tabs.

2.2 Upload and configure the reference database

2.2.1 Prepare the reference workbook

Prepare the reference database as a Microsoft Excel workbook in *.xlsx* format (Figure 6). The workbook should contain one worksheet. If additional worksheets are present, *massMatchR* reads only the first one. The first row must contain the column headers. At minimum, the workbook must include the following columns:

- **Names:** A label for each reference entry, such as a glycan composition (for example, H5N4F1). If labels are not available, include the column header and leave the corresponding data cells blank.
- ***m/z***: Numeric theoretical or expected *m/z* values used for matching . The values may represent unmodified analytes or chemically modified forms, such as permethylated glycans.

	A	B	C
1	Names	m/z	perMe
2	H3N2	910,3278	1171,5831
3	H3N2F1	1056,3857	1345,6723
4	H4N2	1072,3806	1375,6829
5	H3N3	1113,4072	1416,7094
6	H4N2F1	1218,4385	1549,7721
7	H5N2	1234,4334	1579,7826
8	H3N3F1	1259,4651	1590,7986
9	H4N3	1275,4600	1620,8091
10	H3N4	1316,4865	1661,8357

Figure 6. Example structure of a reference workbook.

2.2.2 Upload the reference workbook

Upload the reference workbook by clicking Browse or by dragging and dropping the file onto the upload field in the sidebar panel (Figure 7A).

When the upload is complete, the Pair from columns box lists every detected column together with its index number (Figure 7B). Use these indices in the Names, *m/z*, and Images selectors. After you select the reference *m/z* column, rows with missing *m/z* values are excluded. For matching, the selected values are rounded automatically to four decimal places, and duplicate rounded values are collapsed by retaining the first occurrence.

After uploading the workbook, use the three numeric selectors to map the reference columns:

1. Names: The index of the column containing the labels displayed in tables and plots. The selected column may contain blank values if labels are unavailable.
2. *m/z*: The index of the theoretical or expected *m/z* column used for matching the sample peaks.
3. Images: The index of the column containing the identifiers used to retrieve structure images.

A single reference workbook can contain multiple sets of theoretical *m/z* values corresponding to different analyte forms, such as unmodified, permethylated, or otherwise chemically modified forms. Select the *m/z* column that corresponds to the sample preparation and measurement method. Thanks to this, there is no need to create multiple reference databases with different mass modifications. Also, this structure allows you to match the sample data with the reference database *e.g.* via permethylated *m/z*, and pair the glycan structures from unmodified *m/z* values.

2.2.3 Example settings

The example reference workbook is based on the values retrieved from Kronewitter *et al.*, 2009[6] and contains the three column: Names, *m/z* and perMe. The perMe column contains theoretically calculated permethylated m/z values. For the provided example dataset, use the settings shown in Figure 7C:

1. Names: 1
2. *m/z*: 3
3. Images: 3

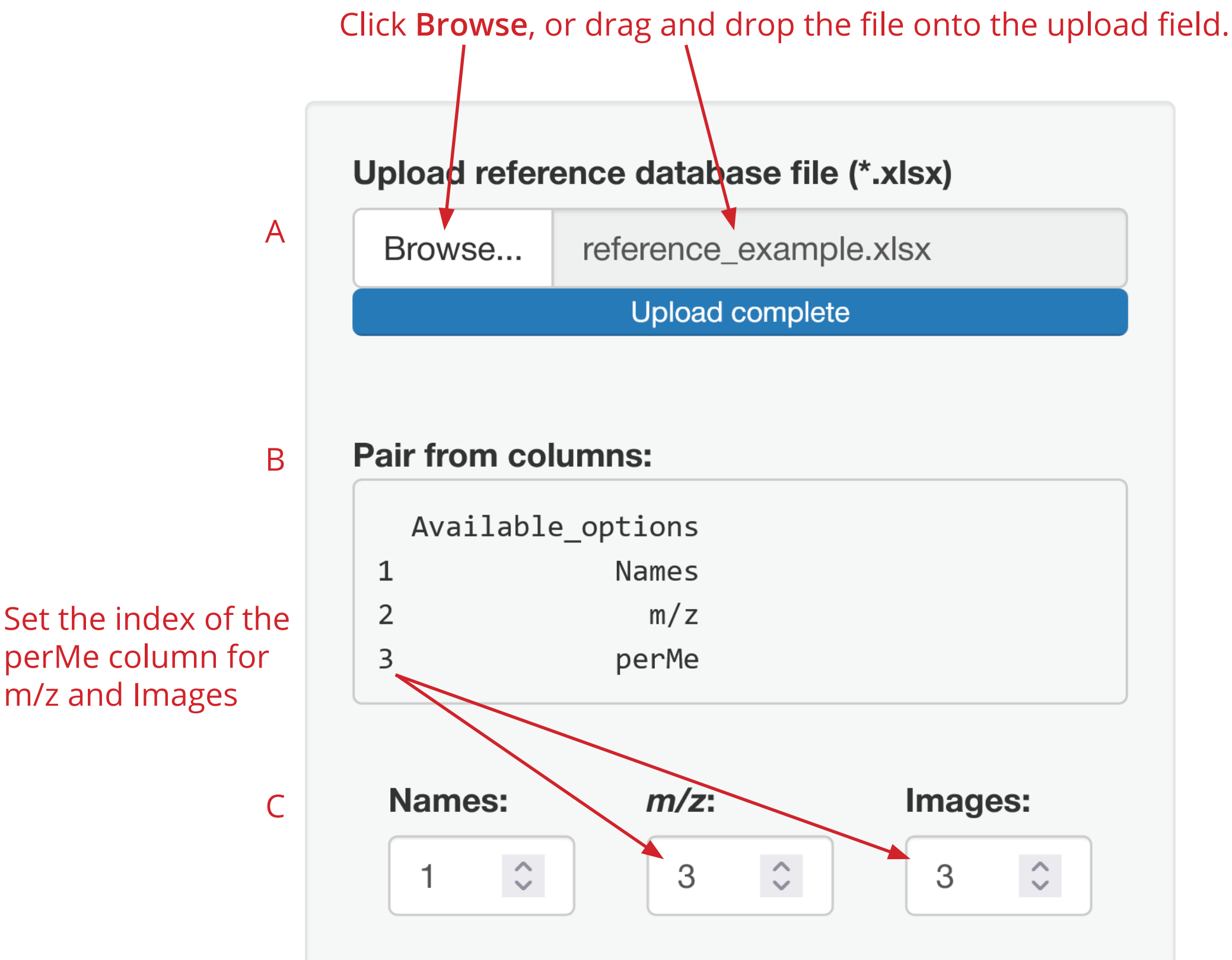

Figure 7. The setup for the uploaded the reference database file. (A) Upload field for the reference workbook. (B) Column indices detected in the uploaded reference workbook. (C) Reference-column settings for the example workbook.

Important notes

- The *m/z* selector is set to column 3 (perMe) because the provided example sample data from Kodrikova *et al.*, 2023[7] contains measurements of permethylated glycans.
- The Images selector is also set to column 3 because the hosted version of *massMatchR* uses the permethylated theoretical *m/z* values as identifiers for the corresponding structure images.
- The displayed structures are theoretical candidate structures. They should not be interpreted as experimentally confirmed or biologically validated glycan assignments.
- The hosted application can display only the structure images available in its configured image library. To use a different image set or identifier scheme, install and configure *massMatchR* locally.

[6] https://analyticalsciencejournals.onlinelibrary.wiley.com/doi/10.1002/pmic.200800760
[7] https://www.mdpi.com/2227-9059/11/2/580

2.3 Explore reference data

Before uploading the sample workbook, open the Explore reference data tab to verify the selected reference columns (Figure 8). The tab displays a preview containing three fields:

- **Name:** Labels from the selected Names column.
- ***m/z:*** Values from the selected reference *m/z* column.
- **Structure:** The structure image associated with the selected Images identifier. If no corresponding image is available, NA is displayed. Hover over an available image to enlarge it.

Use this preview to confirm that the reference workbook was read correctly and that the selected names, mass values, and structure images correspond to one another. If any field is incorrect, return to the sidebar panel and correct the relevant column index before uploading the sample data.

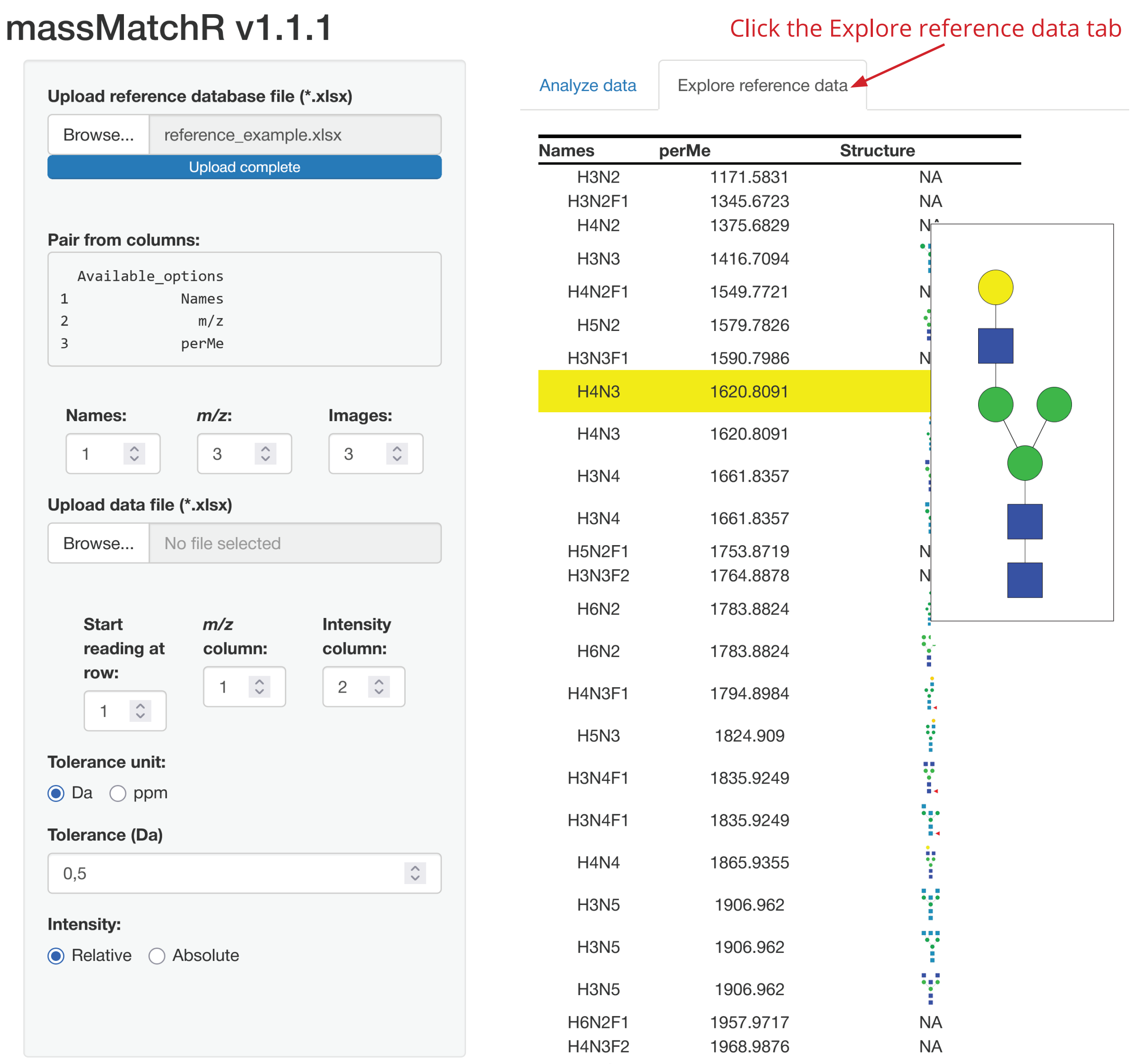

Figure 8. The Explore reference data tab. Hover over an available image to enlarge it.

2.4 Upload and configure the sample data

2.4.1 Prepare the sample workbook

Prepare the sample data as a Microsoft Excel workbook in *.xlsx* format, with each sample stored in a separate worksheet (Figure 9). All worksheets must use the same column order and the same header-row position. They may contain additional columns, but the measured *m/z* and intensity columns must appear in the same positions in every worksheet. Each worksheet must contain at least:

- *m/z*: Measured peak values.
- Intensity: The measured intensity corresponding to each peak.

Worksheet names are automatically used as sample names in the results. Use short, descriptive names that clearly identify the samples.

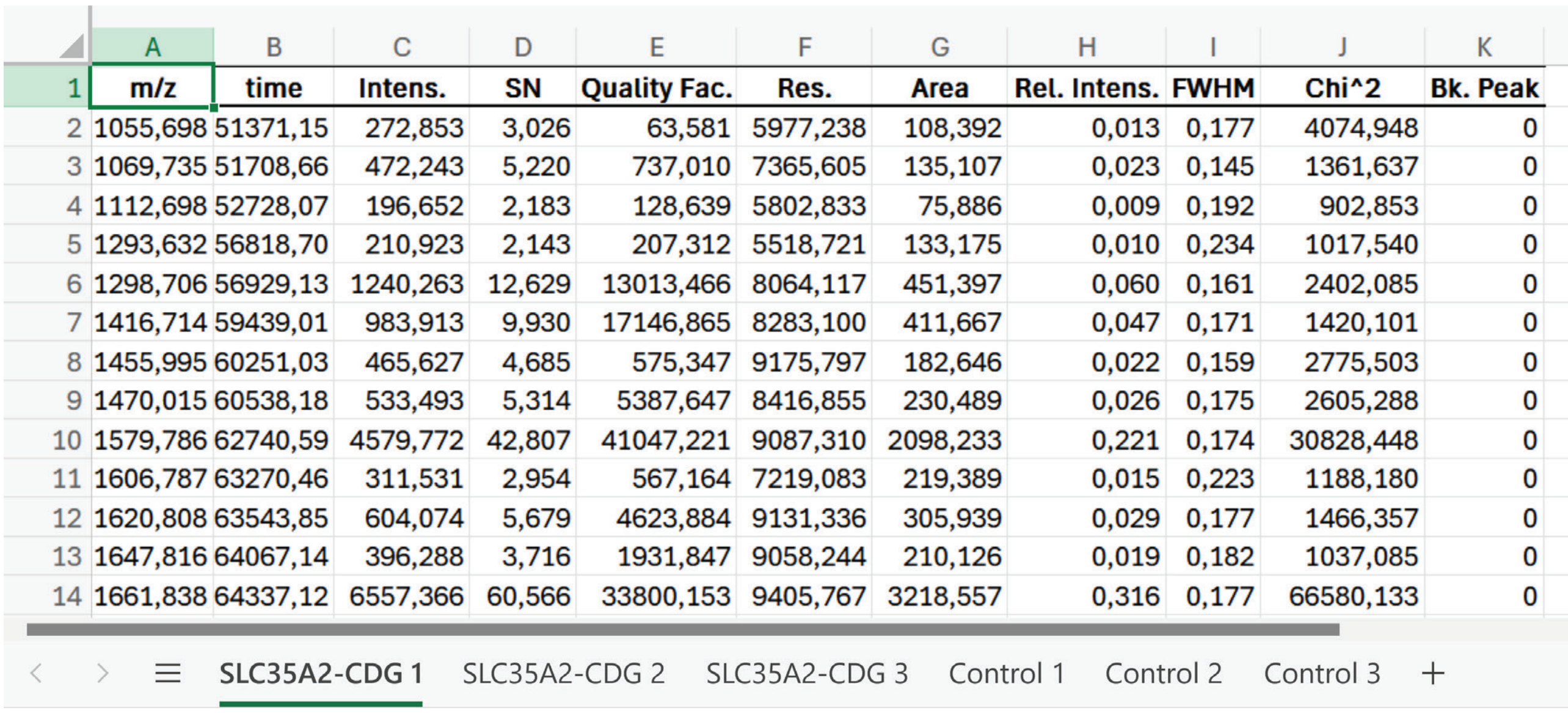

	A	B	C	D	E	F	G	H	I	J	K
1	m/z	time	Intens.	SN	Quality Fac.	Res.	Area	Rel. Intens.	FWHM	Chi^2	Bk. Peak
2	1055,698	51371,15	272,853	3,026	63,581	5977,238	108,392	0,013	0,177	4074,948	0
3	1069,735	51708,66	472,243	5,220	737,010	7365,605	135,107	0,023	0,145	1361,637	0
4	1112,698	52728,07	196,652	2,183	128,639	5802,833	75,886	0,009	0,192	902,853	0
5	1293,632	56818,70	210,923	2,143	207,312	5518,721	133,175	0,010	0,234	1017,540	0
6	1298,706	56929,13	1240,263	12,629	13013,466	8064,117	451,397	0,060	0,161	2402,085	0
7	1416,714	59439,01	983,913	9,930	17146,865	8283,100	411,667	0,047	0,171	1420,101	0
8	1455,995	60251,03	465,627	4,685	575,347	9175,797	182,646	0,022	0,159	2775,503	0
9	1470,015	60538,18	533,493	5,314	5387,647	8416,855	230,489	0,026	0,175	2605,288	0
10	1579,786	62740,59	4579,772	42,807	41047,221	9087,310	2098,233	0,221	0,174	30828,448	0
11	1606,787	63270,46	311,531	2,954	567,164	7219,083	219,389	0,015	0,223	1188,180	0
12	1620,808	63543,85	604,074	5,679	4623,884	9131,336	305,939	0,029	0,177	1466,357	0
13	1647,816	64067,14	396,288	3,716	1931,847	9058,244	210,126	0,019	0,182	1037,085	0
14	1661,838	64337,12	6557,366	60,566	33800,153	9405,767	3218,557	0,316	0,177	66580,133	0

Figure 9. Example multi-worksheet sample workbook.

2.4.2 Upload the sample workbook

Upload the sample workbook by clicking Browse or by dragging and dropping the file onto the Upload data file field in the sidebar panel (Figure 10A).

2.4.3 Configure the sample columns

The following reading options are applied to every worksheet in the sample workbook:

- Start reading at row: The row number containing the column headers.
- *m/z* column: The index of the column containing the measured *m/z* values.
- Intensity column: The index of the column containing the measured intensity values.

This configuration allows data exported from other software to be imported without rearranging the original columns, provided that every worksheet uses the same layout.

2.4.4 Example settings

For the provided sample_example.xlsx workbook, the headers are in row 1, the measured *m/z* values are in column 1, and the intensity values are in column 3. Use the settings shown in Figure 10B:

1. Start reading at row: 1
2. *m/z* column: 1
3. Intensity column: 3

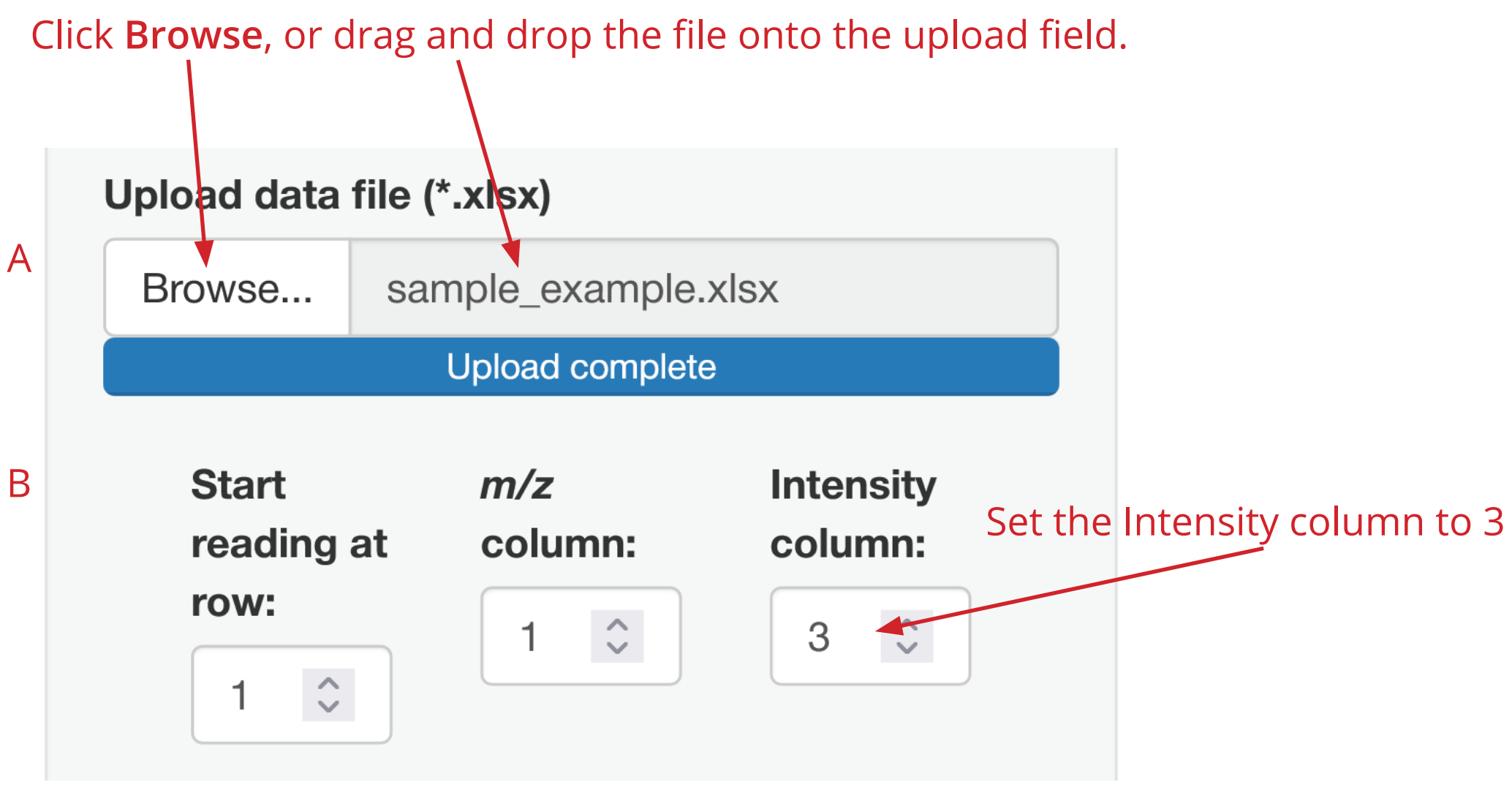

Figure 10. Sample-workbook upload and reading settings: (A) upload field; (B) settings used for the example workbook.

2.5 Matching

After the reference database and the sample workbook are uploaded, *massMatchR* automatically analyzes the first worksheet because Number of samples is initially set to 1. The application compares the measured *m/z* values with the selected reference *m/z* values using the current tolerance unit and tolerance value. With the default intensity mode, matched intensities are reported as relative percentages. The results are displayed in the Samples tab of the main panel (Figure 11).

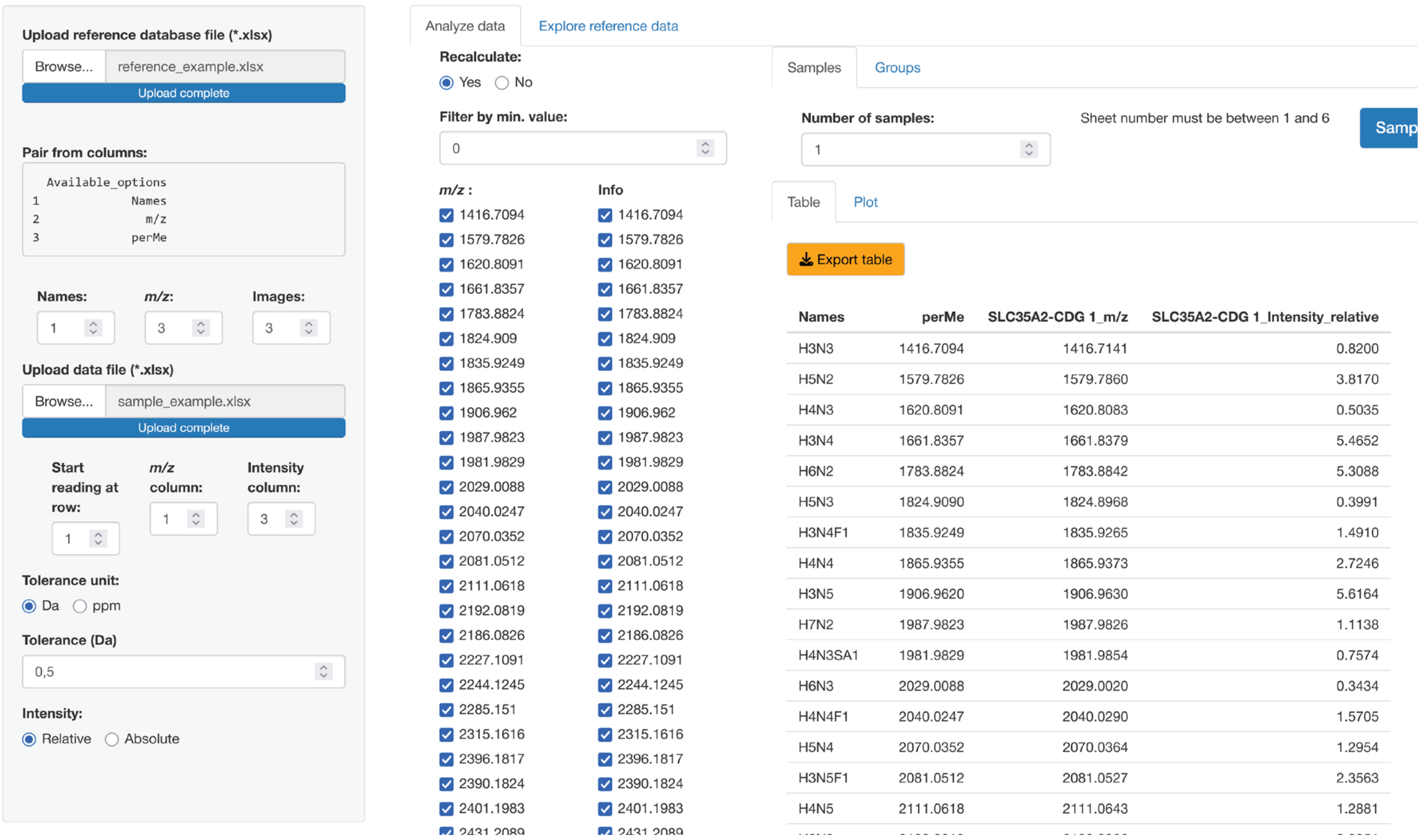

Figure 11. Results after the initial matching of the first sample.

The examples in the following sections use the default matching settings unless otherwise stated. You can change the tolerance and intensity settings in the sidebar panel (Figure 12).

2.5.1 Tolerance unit

Select the unit used to calculate the permitted difference between a reference *m/z* value and a measured peak:

- **Da:** Uses a fixed absolute difference on either side of each reference value. For example, with a tolerance of 0.5 Da, a reference value of 1000.0 can match measured peaks from 999.5 to 1000.5.
- **ppm:** Uses a mass-dependent relative tolerance. The ppm difference expresses the deviation between the measured and reference *m/z* values relative to the reference *m/z*, in parts per million. At 20 ppm, a reference *m/z* of 1000.0000 has an absolute tolerance of ±0.0200 Da, so measured peaks from 999.9800 to 1000.0200 are accepted. A reference *m/z* of 2000.0000 has an absolute tolerance of ±0.0400 Da, so measured peaks from 1999.9600 to 2000.0400 are accepted. Therefore, the same ppm tolerance corresponds to a wider absolute matching window at higher *m/z* values.

2.5.2 Tolerance

Enter the maximum permitted difference between the reference and measured *m/z* values. The default tolerance is 0.5 Da. When ppm is selected, the default is 20 ppm. A smaller value produces stricter matching, whereas a larger value accepts peaks farther from the reference value.

2.5.3 Intensity

Select how the intensity of each matched peak is reported:

- **Relative:** Each matched intensity is divided by the sum of all matched intensities in the same sample and multiplied by 100. The result is displayed as a percentage.
- **Absolute:** The original measured intensity of the selected peak is reported without normalization.

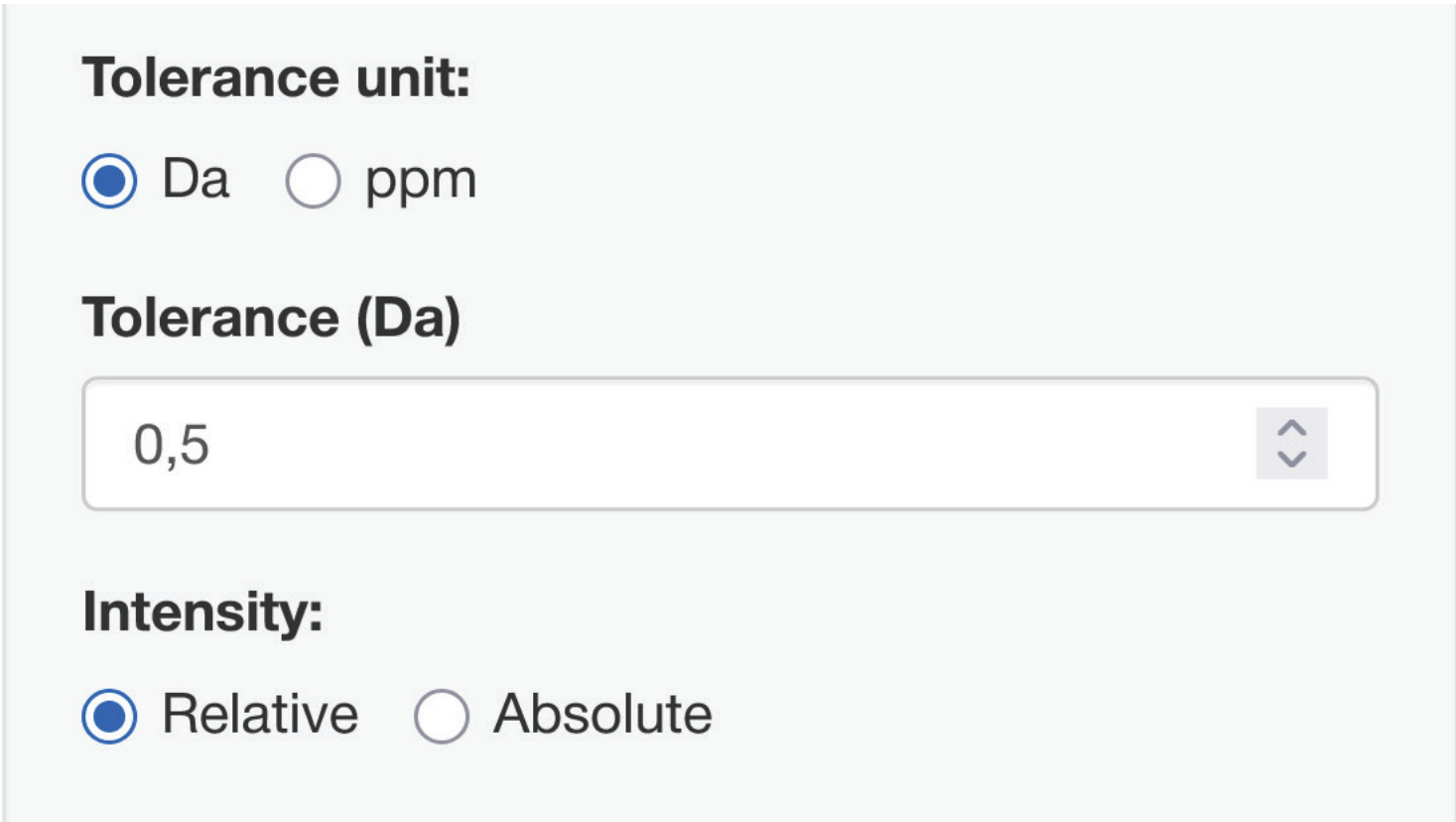

Figure 12. Tolerance and intensity controls.

3 Sample analysis

The Samples tab contains the results for individual samples. It is divided into a Table tab and a Plot tab. Controls above these tabs allow you to select the samples to analyze, rename them, and assign colors to them.

3.1 Selecting the number of samples

After a sample workbook is uploaded, *massMatchR* counts its worksheets and displays the permitted range next to Number of samples. For example, if the workbook contains six worksheets, the displayed message states that the number of samples must be between 1 and 6 (Figure 13).

massMatchR reads the worksheets consecutively from the beginning of the workbook. When the sample workbook is first uploaded, Number of samples is set to 1. Increase this value to include additional worksheets. Changing the number of samples causes *massMatchR* to read the selected worksheets again, repeat the matching procedure, and update the result tables, plots, sample options, and group-assignment controls.

The worksheet names are used as the initial sample names. Therefore, worksheet names should be unique and should clearly identify the samples.

Currently it is not possible to select non-consecutive worksheets. To exclude a worksheet located between two worksheets that should be analyzed, rearrange the workbook before uploading it or create a new workbook containing only the required samples.

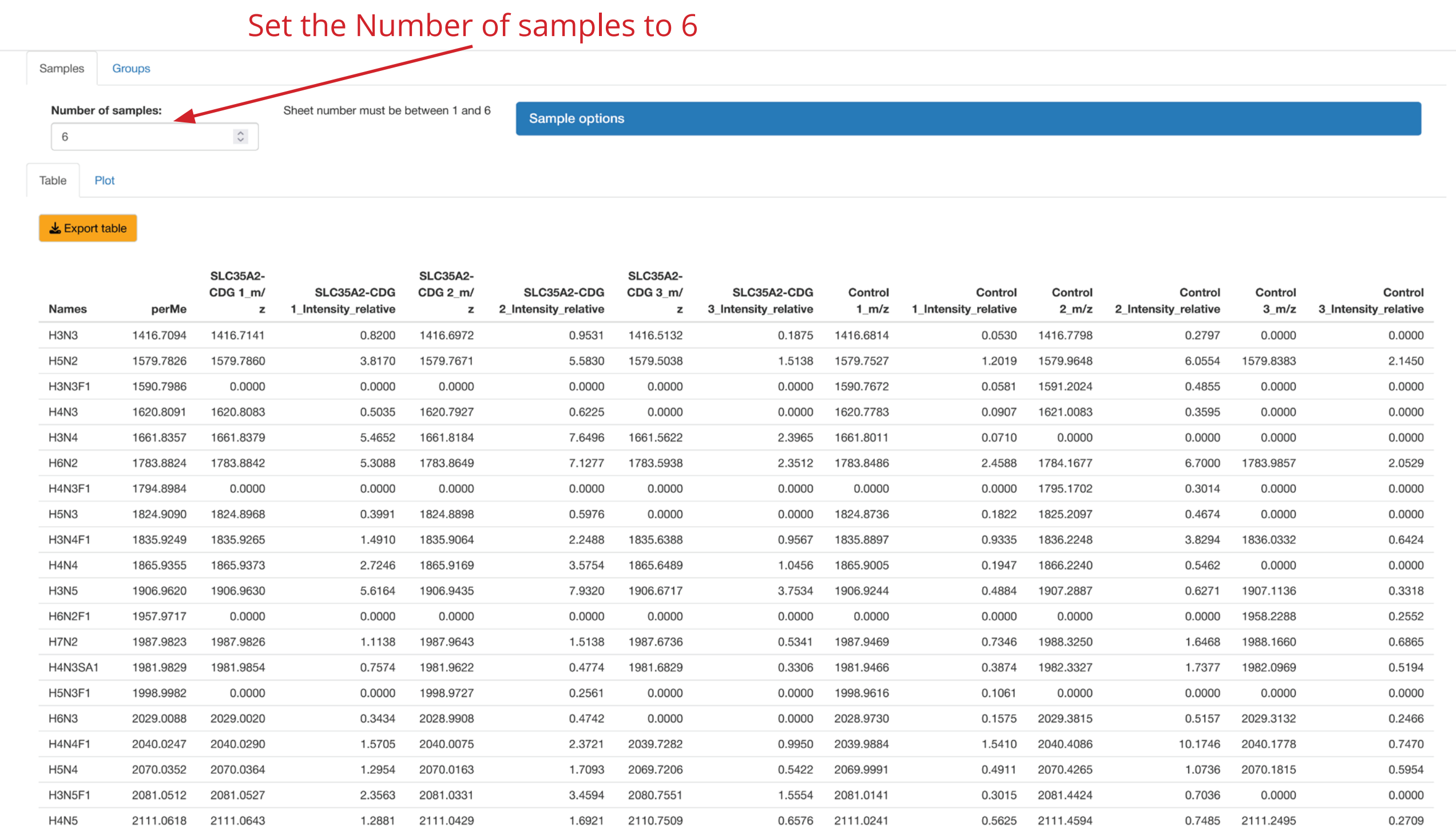

Names	perMe	SLC35A2-CDG 1_m/z	SLC35A2-CDG 1_Intensity_relative	SLC35A2-CDG 2_m/z	SLC35A2-CDG 2_Intensity_relative	SLC35A2-CDG 3_m/z	SLC35A2-CDG 3_Intensity_relative	Control 1_m/z	Control 1_Intensity_relative	Control 2_m/z	Control 2_Intensity_relative	Control 3_m/z	Control 3_Intensity_relative
H3N3	1416.7094	1416.7141	0.8200	1416.6972	0.9531	1416.5132	0.1875	1416.6814	0.0530	1416.7798	0.2797	0.0000	0.0000
H5N2	1579.7826	1579.7860	3.8170	1579.7671	5.5830	1579.5038	1.5138	1579.7527	1.2019	1579.9648	6.0554	1579.8383	2.1450
H3N3F1	1590.7986	0.0000	0.0000	0.0000	0.0000	0.0000	0.0000	1590.7672	0.0581	1591.2024	0.4855	0.0000	0.0000
H4N3	1620.8091	1620.8083	0.5035	1620.7927	0.6225	0.0000	0.0000	1620.7783	0.0907	1621.0083	0.3595	0.0000	0.0000
H3N4	1661.8357	1661.8379	5.4652	1661.8184	7.6496	1661.5622	2.3965	1661.8011	0.0710	0.0000	0.0000	0.0000	0.0000
H6N2	1783.8824	1783.8842	5.3088	1783.8649	7.1277	1783.5938	2.3512	1783.8486	2.4588	1784.1677	6.7000	1783.9857	2.0529
H4N3F1	1794.8984	0.0000	0.0000	0.0000	0.0000	0.0000	0.0000	0.0000	0.0000	1795.1702	0.3014	0.0000	0.0000
H5N3	1824.9090	1824.8968	0.3991	1824.8898	0.5976	0.0000	0.0000	1824.8736	0.1822	1825.2097	0.4674	0.0000	0.0000
H3N4F1	1835.9249	1835.9265	1.4910	1835.9064	2.2488	1835.6388	0.9567	1835.8897	0.9335	1836.2248	3.8294	1836.0332	0.6424
H4N4	1865.9355	1865.9373	2.7246	1865.9169	3.5754	1865.6489	1.0456	1865.9005	0.1947	1866.2240	0.5462	0.0000	0.0000
H3N5	1906.9620	1906.9630	5.6164	1906.9435	7.9320	1906.6717	3.7534	1906.9244	0.4884	1907.2887	0.6271	1907.1136	0.3318
H6N2F1	1957.9717	0.0000	0.0000	0.0000	0.0000	0.0000	0.0000	0.0000	0.0000	0.0000	0.0000	1958.2288	0.2552
H7N2	1987.9823	1987.9826	1.1138	1987.9643	1.5138	1987.6736	0.5341	1987.9469	0.7346	1988.3250	1.6468	1988.1660	0.6865
H4N3SA1	1981.9829	1981.9854	0.7574	1981.9622	0.4774	1981.6829	0.3306	1981.9466	0.3874	1982.3327	1.7377	1982.0969	0.5194
H5N3F1	1998.9982	0.0000	0.0000	1998.9727	0.2561	0.0000	0.0000	1998.9616	0.1061	0.0000	0.0000	0.0000	0.0000
H6N3	2029.0088	2029.0020	0.3434	2028.9908	0.4742	0.0000	0.0000	2028.9730	0.1575	2029.3815	0.5157	2029.3132	0.2466
H4N4F1	2040.0247	2040.0290	1.5705	2040.0075	2.3721	2039.7282	0.9950	2039.9884	1.5410	2040.4086	10.1746	2040.1778	0.7470
H5N4	2070.0352	2070.0364	1.2954	2070.0163	1.7093	2069.7206	0.5422	2069.9991	0.4911	2070.4265	1.0736	2070.1815	0.5954
H3N5F1	2081.0512	2081.0527	2.3563	2081.0331	3.4594	2080.7551	1.5554	2081.0141	0.3015	2081.4424	0.7036	0.0000	0.0000
H4N5	2111.0618	2111.0643	1.2881	2111.0429	1.6921	2110.7509	0.6576	2111.0241	0.5625	2111.4594	0.7485	2111.2495	0.2709

Figure 13. Selecting the number of samples. The message next to the input shows the number of worksheets available in the uploaded workbook.

3.2 Renaming samples

Open the Sample options panel to view the Sample names and Sample colors controls.

The Sample names field is automatically populated with the names of the selected worksheets. Individual names are separated by semicolons. After uploading the example sample data you should see: SLC35A2-CDG 1;SLC35A2-CDG 2;SLC35A2-CDG 3;Control 1;Control 2;Control 3 (Figure 14A).

To rename the samples:

1. Edit the names in the Sample names field.
2. Keep the names in the same order as the worksheets.
3. Separate consecutive names with semicolons.
4. Click Rename sample(s).

Enter one name for every selected sample. Do not use semicolons as part of a sample name because the application uses them as separators. Spaces at the beginning or end of a name are removed automatically. The new names are used throughout subsequent tables, plots, group controls, and export, renaming a sample changes only its displayed label, It does not modify the corresponding worksheet or the uploaded Excel workbook.

Click Reset sample name(s) to restore the original worksheet names.

To test this functionality, type into the Sample names field: Patient 1;Patient 2;Patient 3;Control 1;Control 2;Control 3 and click Rename sample(s). You should see the new sample names in the Sample colors controls and also in the result table (Figure 14B).

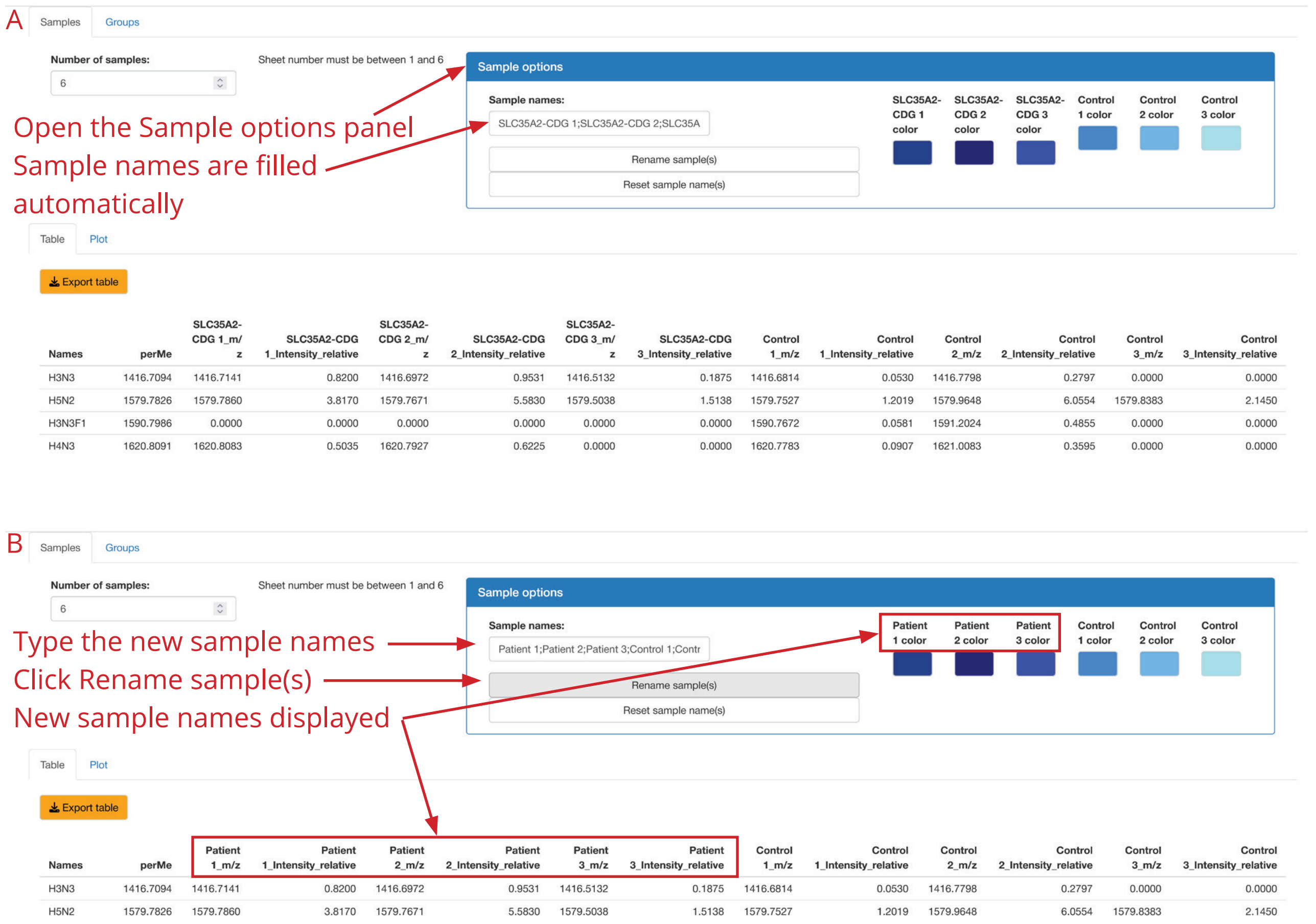

Figure 14. Renaming samples in the Sample options panel. Sample names are entered in worksheet order and separated by semicolons. (A) The Sample names field is automatically populated with the names of the selected worksheets. (B) The Sample names after renaming.

3.3 Setting sample colors

The Sample options panel provides one color selector for each sample. The label above each selector contains the current sample name (Figure 15A). Click a color field to open the color picker, and select the color used to represent that sample in the sample plot. The plot is updated after the color is changed (Figure 15B).

Sample colors affect only the graphical representation of the results. They do not affect matching, intensity calculations, tables, group assignments, statistical tests, or exported tabular data.

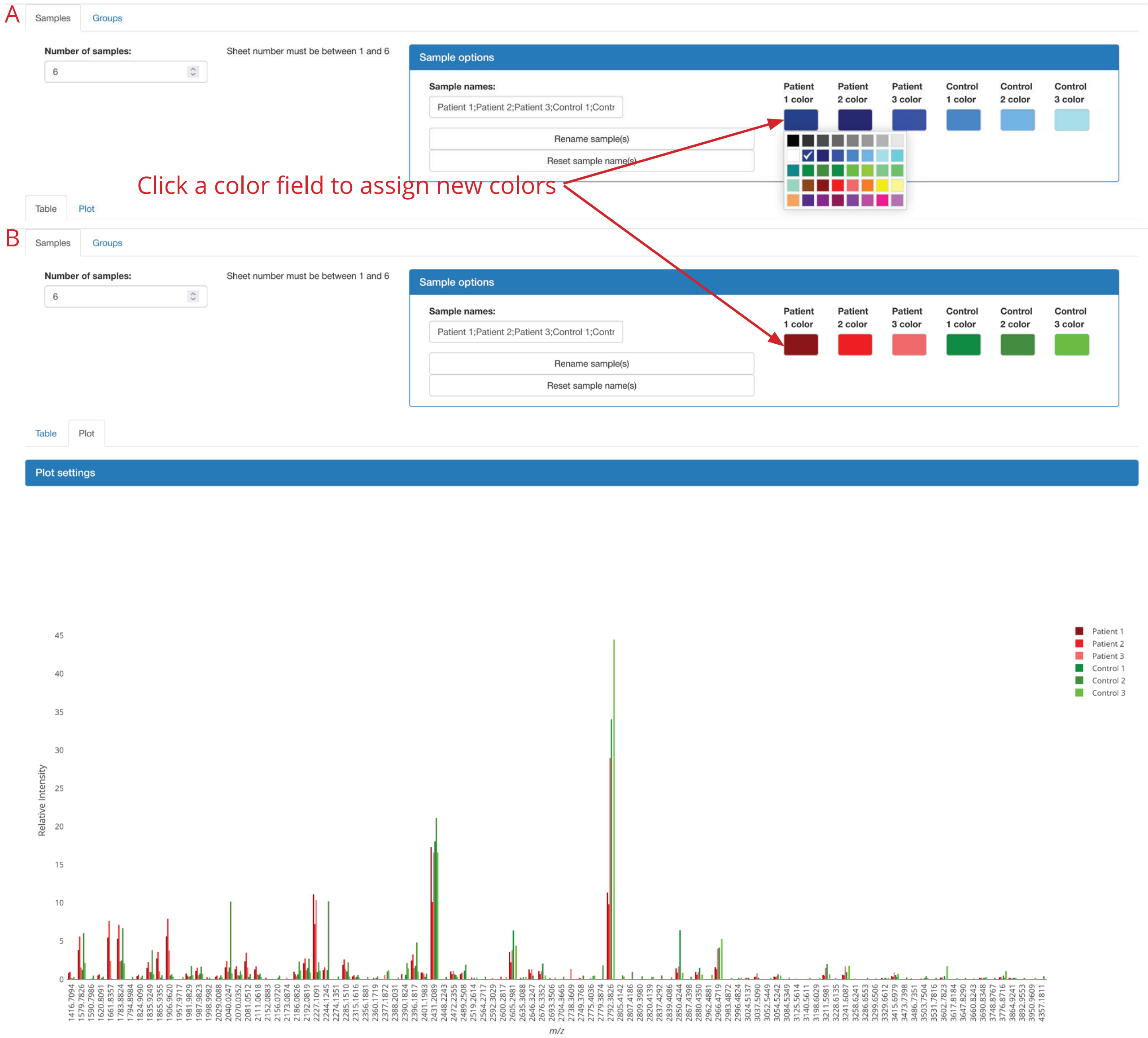

Figure 15. Sample-color selectors. Each selected sample has an independent plot color. (A) The default colors assigned to each sample. (B) Custom colors assigned to samples.

3.4 Sample results table

Open the Table tab under Samples to inspect the matched results (Figure 16).

The first columns identify the reference entry:

- Names: Value from the selected reference Names column.
- *m/z*: Selected theoretical or expected reference *m/z* value.
- Two additional columns are displayed for each selected sample: the measured *m/z* value of the closest peak that satisfied the tolerance criterion; the corresponding relative or absolute intensity.

If no measured peak satisfied the selected tolerance for a sample, its matched *m/z* and intensity are recorded as 0. In Relative mode, the intensity columns contain normalized percentages. Their values depend on the Recalculate setting. In Absolute mode, the columns contain the original measured intensities from the sample workbook. The table shown in the interface is rounded to four decimal places for readability.

3.4.1 Exporting the sample table

Click Export table (Figure 16) to download the sample results as sample_table.xlsx. The exported table contains the same scientifically relevant columns as the displayed table (Figure 17).

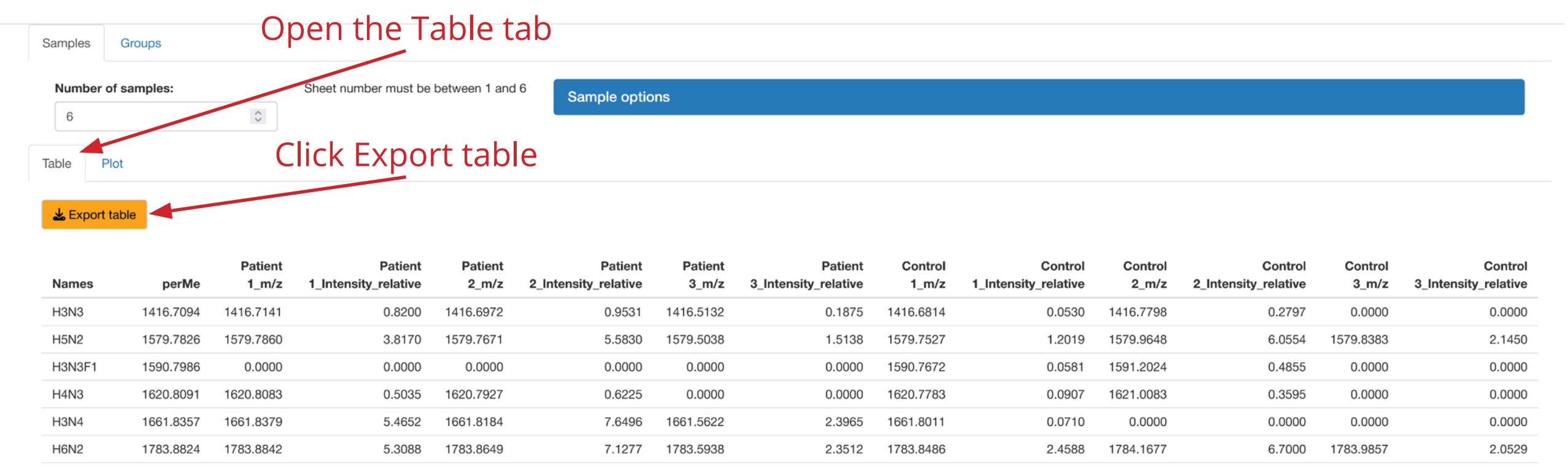

Names	perMe	Patient 1_m/z	Patient 1_Intensity_relative	Patient 2_m/z	Patient 2_Intensity_relative	Patient 3_m/z	Patient 3_Intensity_relative	Control 1_m/z	Control 1_Intensity_relative	Control 2_m/z	Control 2_Intensity_relative	Control 3_m/z	Control 3_Intensity_relative
H3N3	1416.7094	1416.7141	0.8200	1416.6972	0.9531	1416.5132	0.1875	1416.6814	0.0530	1416.7798	0.2797	0.0000	0.0000
H5N2	1579.7826	1579.7860	3.8170	1579.7671	5.5830	1579.5038	1.5138	1579.7527	1.2019	1579.9648	6.0554	1579.8383	2.1450
H3N3F1	1590.7986	0.0000	0.0000	0.0000	0.0000	0.0000	0.0000	1590.7672	0.0581	1591.2024	0.4855	0.0000	0.0000
H4N3	1620.8091	1620.8083	0.5035	1620.7927	0.6225	0.0000	0.0000	1620.7783	0.0907	1621.0083	0.3595	0.0000	0.0000
H3N4	1661.8357	1661.8379	5.4652	1661.8184	7.6496	1661.5622	2.3965	1661.8011	0.0710	0.0000	0.0000	0.0000	0.0000
H6N2	1783.8824	1783.8842	5.3088	1783.8649	7.1277	1783.5938	2.3512	1783.8486	2.4588	1784.1677	6.7000	1783.9857	2.0529

Figure 16. Sample results table containing the reference information, matched measured *m/z*, and sample intensities.

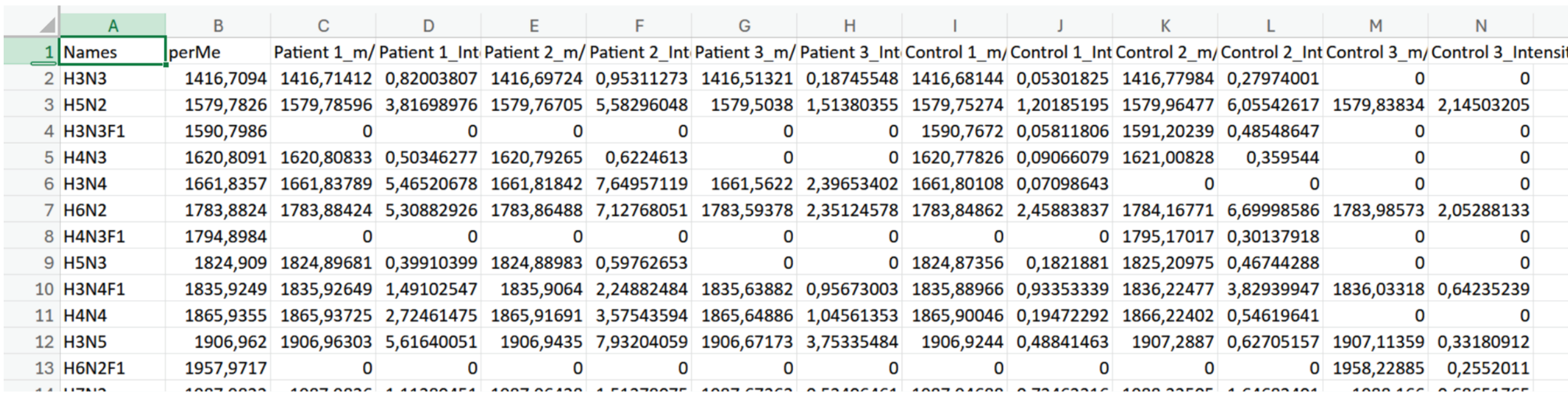

	A	B	C	D	E	F	G	H	I	J	K	L	M	N
1	Names	perMe	Patient 1_m/	Patient 1_Int	Patient 2_m/	Patient 2_Int	Patient 3_m/	Patient 3_Int	Control 1_m/	Control 1_Int	Control 2_m/	Control 2_Int	Control 3_m/	Control 3_Intensit
2	H3N3	1416,7094	1416,71412	0,82003807	1416,69724	0,95311273	1416,51321	0,18745548	1416,68144	0,05301825	1416,77984	0,27974001	0	0
3	H5N2	1579,7826	1579,78596	3,81698976	1579,76705	5,58296048	1579,5038	1,51380355	1579,75274	1,20185195	1579,96477	6,05542617	1579,83834	2,14503205
4	H3N3F1	1590,7986	0	0	0	0	0	0	1590,7672	0,05811806	1591,20239	0,48548647	0	0
5	H4N3	1620,8091	1620,80833	0,50346277	1620,79265	0,6224613	0	0	1620,77826	0,09066079	1621,00828	0,359544	0	0
6	H3N4	1661,8357	1661,83789	5,46520678	1661,81842	7,64957119	1661,5622	2,39653402	1661,80108	0,07098643	0	0	0	0
7	H6N2	1783,8824	1783,88424	5,30882926	1783,86488	7,12768051	1783,59378	2,35124578	1783,84862	2,45883837	1784,16771	6,69998586	1783,98573	2,05288133
8	H4N3F1	1794,8984	0	0	0	0	0	0	0	0	1795,17017	0,30137918	0	0
9	H5N3	1824,909	1824,89681	0,39910399	1824,88983	0,59762653	0	0	1824,87356	0,1821881	1825,20975	0,46744288	0	0
10	H3N4F1	1835,9249	1835,92649	1,49102547	1835,9064	2,24882484	1835,63882	0,95673003	1835,88966	0,93353339	1836,22477	3,82939947	1836,03318	0,64235239
11	H4N4	1865,9355	1865,93725	2,72461475	1865,91691	3,57543594	1865,64886	1,04561353	1865,90046	0,19472292	1866,22402	0,54619641	0	0
12	H3N5	1906,962	1906,96303	5,61640051	1906,9435	7,93204059	1906,67173	3,75335484	1906,9244	0,48841463	1907,2887	0,62705157	1907,11359	0,33180912
13	H6N2F1	1957,9717	0	0	0	0	0	0	0	0	0	0	1958,22885	0,2552011

Figure 17. Example of the sample results exported as an Excel workbook.

3.5 Filtering the matched results

The controls on the left side of the Analyze data panel are shared by the Samples and Groups tabs. They determine which matched reference entries are included, whether relative intensities are recalculated, which values are displayed in plots, and which supplementary annotations appear above the bars.

3.5.1 Selecting and deselecting reference values

After matching is complete, the *m/z* checklist displays the reference *m/z* values for which at least one match was detected in the selected samples. All values are initially selected (Figure 18A). A checked *m/z* value is included in the analysis. Deselecting a value (Figure 18B) removes the corresponding reference entry from:

- the sample table;
- the sample plot;
- group means, standard deviations, and standard errors;
- the group plot;
- statistical tests;
- exported sample, group, and statistical tables.

The Select/Deselect all button at the bottom of the *m/z* list alternates between clearing and selecting the complete list. At least one *m/z* value must remain selected. If all values are deselected, *massMatchR* displays a message asking you to select at least one value.

This filter removes complete reference entries. It is therefore different from the minimum-value plot filter and the Info checklist, which hides individual bars or plot annotations without removing the corresponding data from tables or statistical calculations.

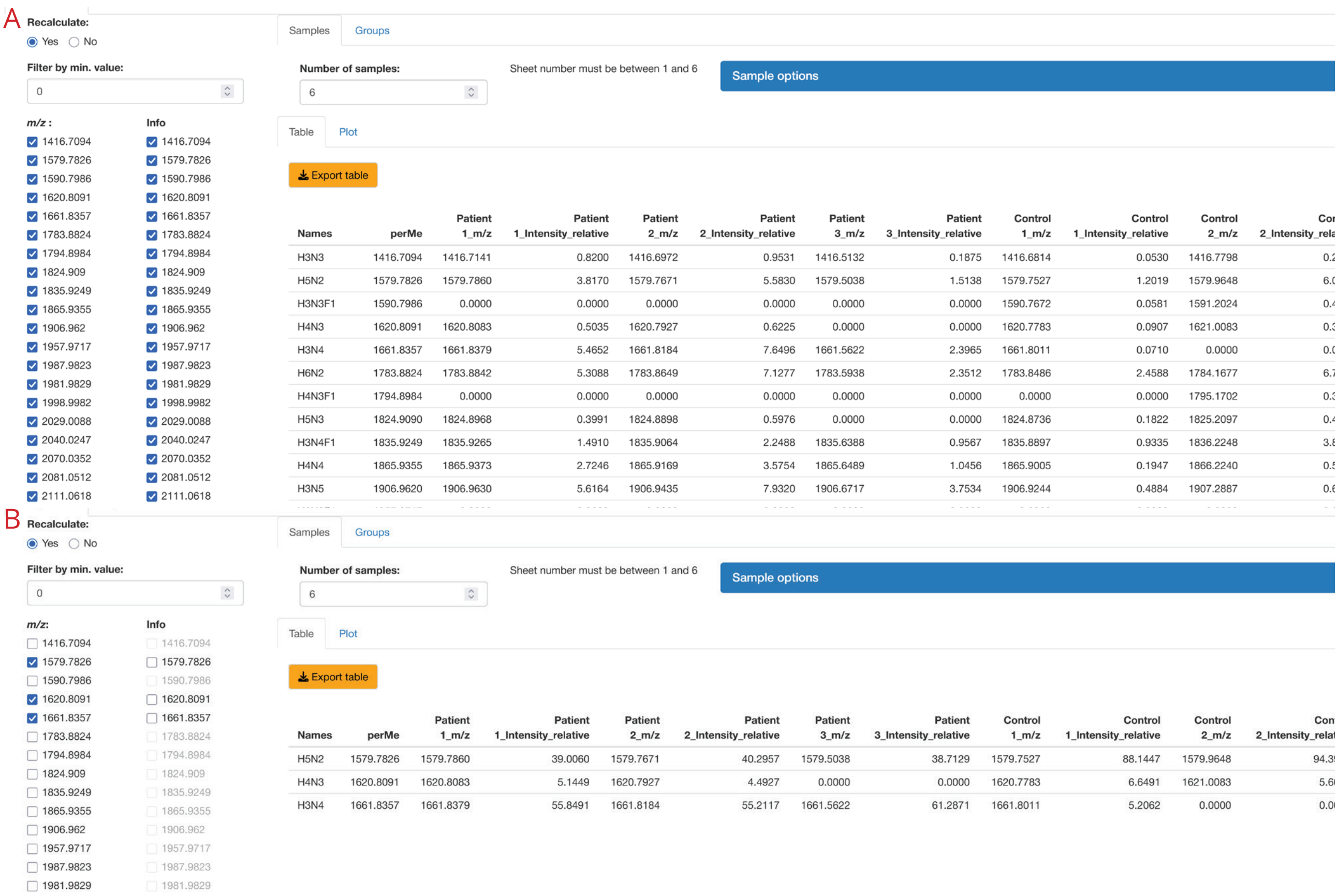

Figure 18. The *m/z* checklist used to include or exclude matched reference entries. (A) Initially all matched values are checked. (B) Deselecting values removes them from further analyses.

3.5.2 Recalculating relative intensities

The Recalculate control determines how relative intensities are handled after one or more *m/z* values have been deselected. This setting affects only Relative intensity mode. It has no effect when Absolute intensity is selected.

When Recalculate: Yes is selected (Figure 19A), relative intensities are recalculated using only the currently selected *m/z* values. For every sample, each selected intensity is divided by the sum of the selected intensities. Consequently, the remaining selected intensities normally sum to 100% within each sample.

When Recalculate is set to No (Figure 19B), relative intensities retain their proportions relative to the complete set of matched peaks. The denominator is calculated before the manual *m/z* filtering is applied.

The recalculation setting affects:

- the sample table;
- group summaries;
- statistical analysis;
- exported tables.

It does not repeat the *m/z* matching procedure; it only changes the normalization of the already matched intensities.

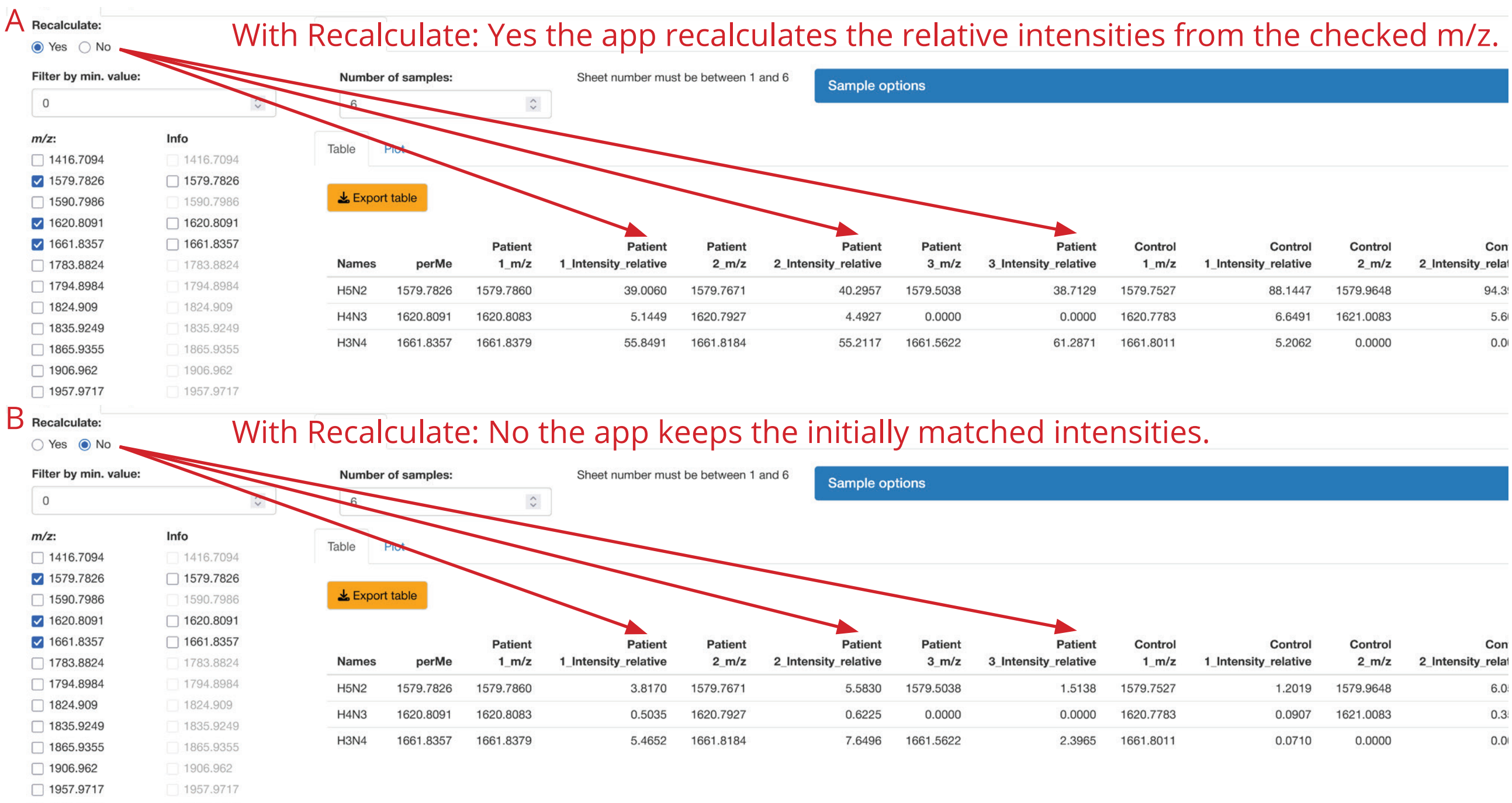

Figure 19. Effect of the Recalculate setting on relative intensities after selected reference values are removed. (A) Recalculate: Yes renormalizes the selected intensities; (B) Recalculate: No preserves their original relative contributions.

For demonstration purposes only we selected the following *m/z*: 1579.7826, 1620.8091, 1661.8357, 1783.8824, 1835.9249, 1865.9355, 1906.962, 1987.9823, 2040.0247, 2070.0352, 2081.0512, 2192.0819, 2227.1091, 2244.1245, 2285.151, 2396.1817, 2390.1824, 2401.1983, 2431.2089, 2605.2981, 2676.3352, 2792.3826, 2850.4244, 2880.435, 2996.4824, 3054.5242, 3211.5981, 3241.6087, 3415.6979, 3602.7823, 3690.8348, 3776.8716 with Recalculate set to Yes.

3.6 Sample plot

Open the Plot tab under Samples to display the matched intensities as a grouped bar plot. Each *m/z* category contains one bar for every selected sample. Sample names are displayed in the legend, and the colors are taken from the Sample options panel (Figure 20).

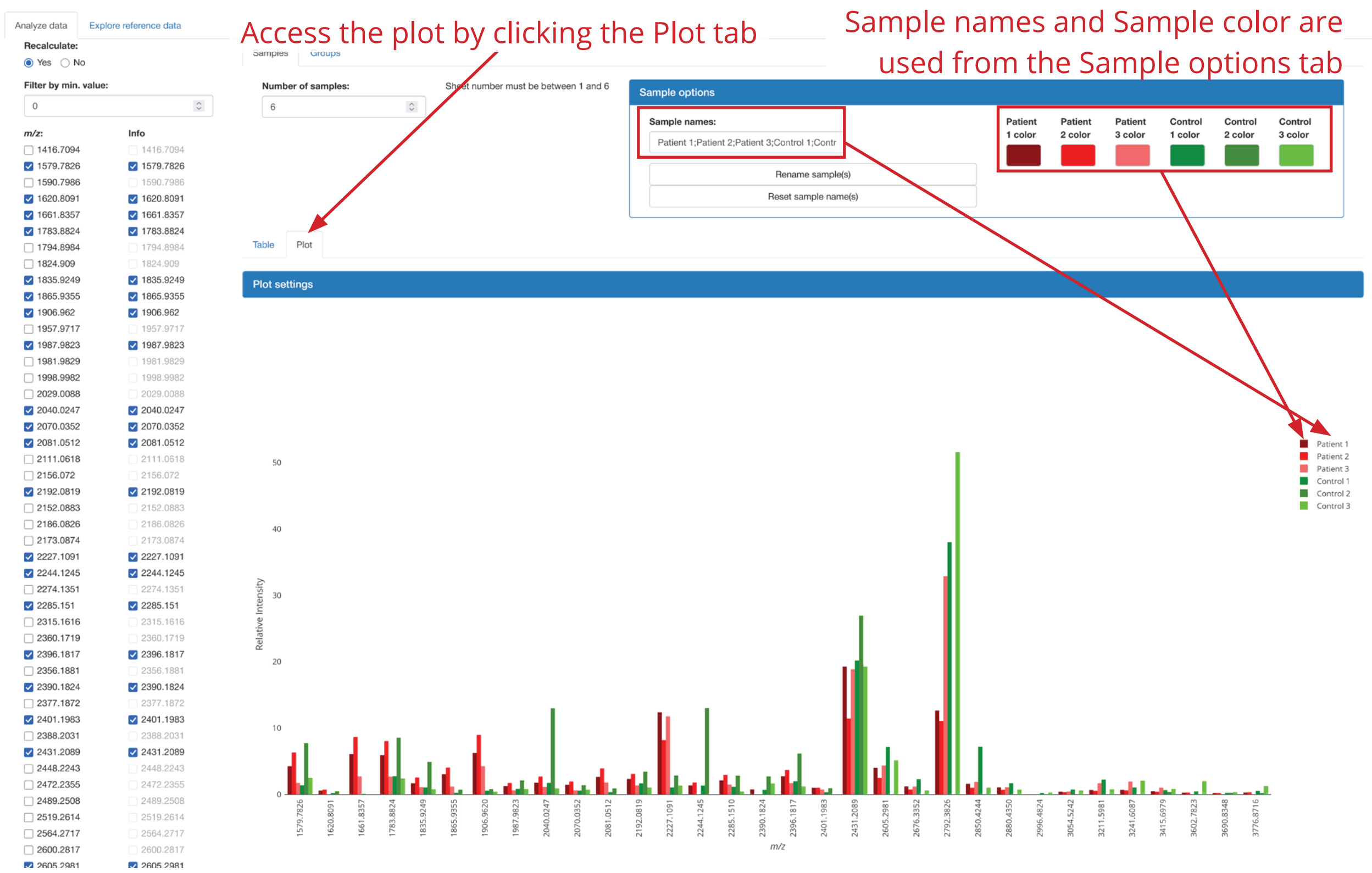

Figure 20. The Plot tab displaying the matched and filtered results.

3.6.1 Filtering the plot by a minimum value

The Filter by min. value input hides bars whose values are below a selected threshold. Enter 0 to disable this plot filter (Figure 20). When a value greater than zero is entered (Figure 21):

- In the sample plot, each sample bar below the threshold is hidden.
- In the group plot, each group-mean bar below the threshold is hidden.
- if every displayed value for an *m/z* category is below the threshold or equal to zero, the complete category is removed from the plot.

Values equal to the threshold remain visible. Only values strictly below it are hidden. The unit of the threshold depends on the selected intensity mode:

- in Relative mode, it is applied to relative-intensity percentages;
- in Absolute mode, it is applied to raw measured intensities.

This is a visualization filter only. It does not change:

- the sample or group tables;
- the selected *m/z* checklist;
- relative-intensity recalculation;
- group statistics;
- exported tabular results.

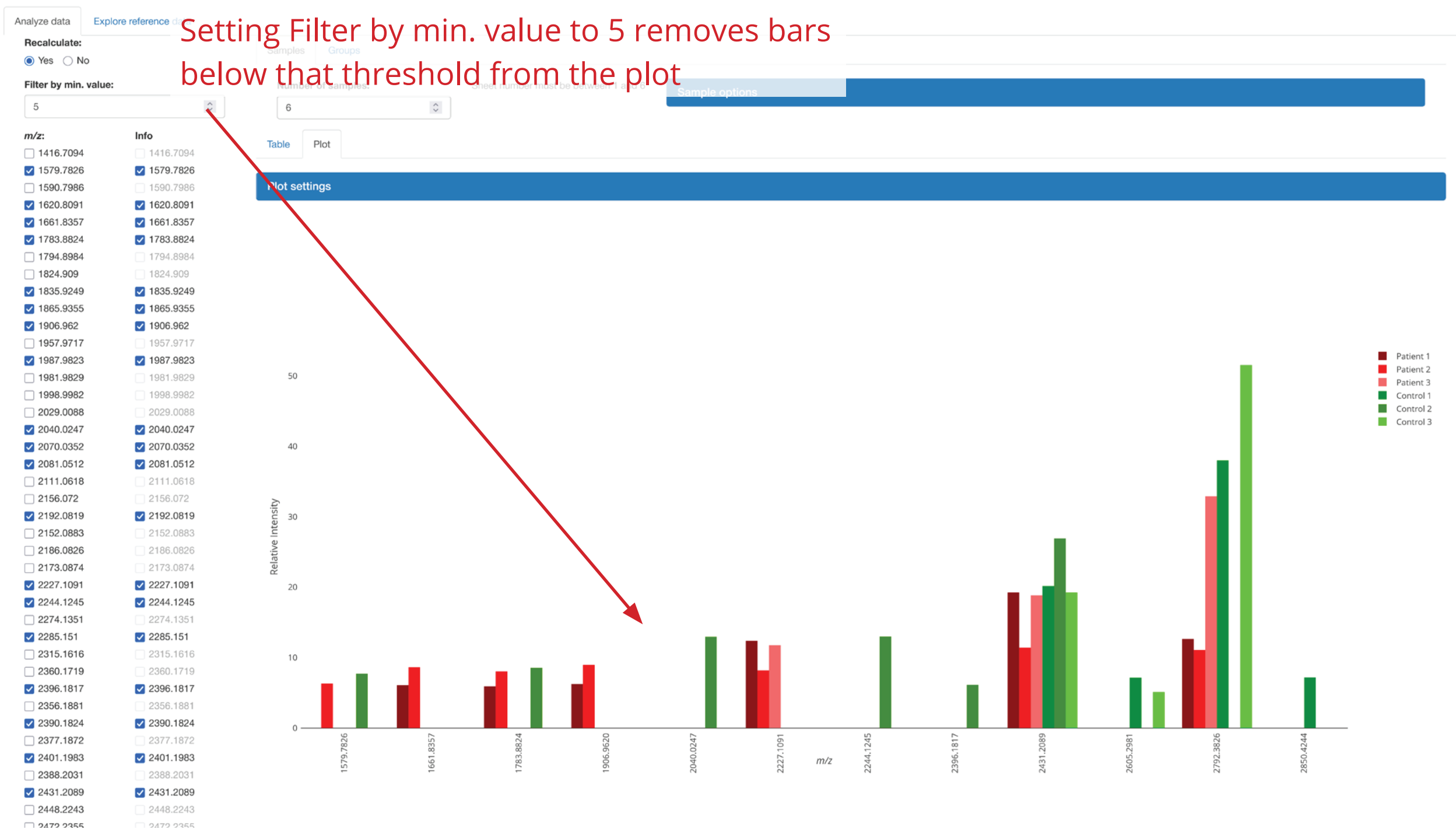

Figure 21. Filtering the sample plot by a minimum displayed value set to 5.

3.6.2 Plot settings

Expand the Plot settings panel to customize the plot (Figure 22). The interface provides controls for the title, axis labels, annotations, and exported image dimensions.

Plot title

Enter the required title in Plot title (e.g., SLC35A2), and then click the Name plot button to apply it. Entering text alone does not update the title until the button is clicked. If the title field is empty when Name plot is clicked, the application uses: *Default plot title*. Use Plot title font size to change the title size.

Axis settings

The following controls change the appearance of the plot axes:

- Plot font rotation: controls the orientation of the *m/z* tick labels on the horizontal axis. Left rotates labels by −90°, Horizontal displays labels horizontally, Right rotates labels by 90°. This setting does not rotate the plot title or axis titles.
- Plot axis label font size: controls the size of the axis titles, i.e. *m/z* on the horizontal axis and Relative Intensity or Absolute Intensity on the vertical axis.
- Plot axis font size: controls the size of the tick labels along both axes.
- Plot axis decimal: specifies the number of decimal places used to display the reference *m/z* values on the horizontal axis. The available range is zero to four decimal places. This setting changes only the displayed labels. It does not alter the stored reference values, the four-decimal matching procedure, or the matching results.

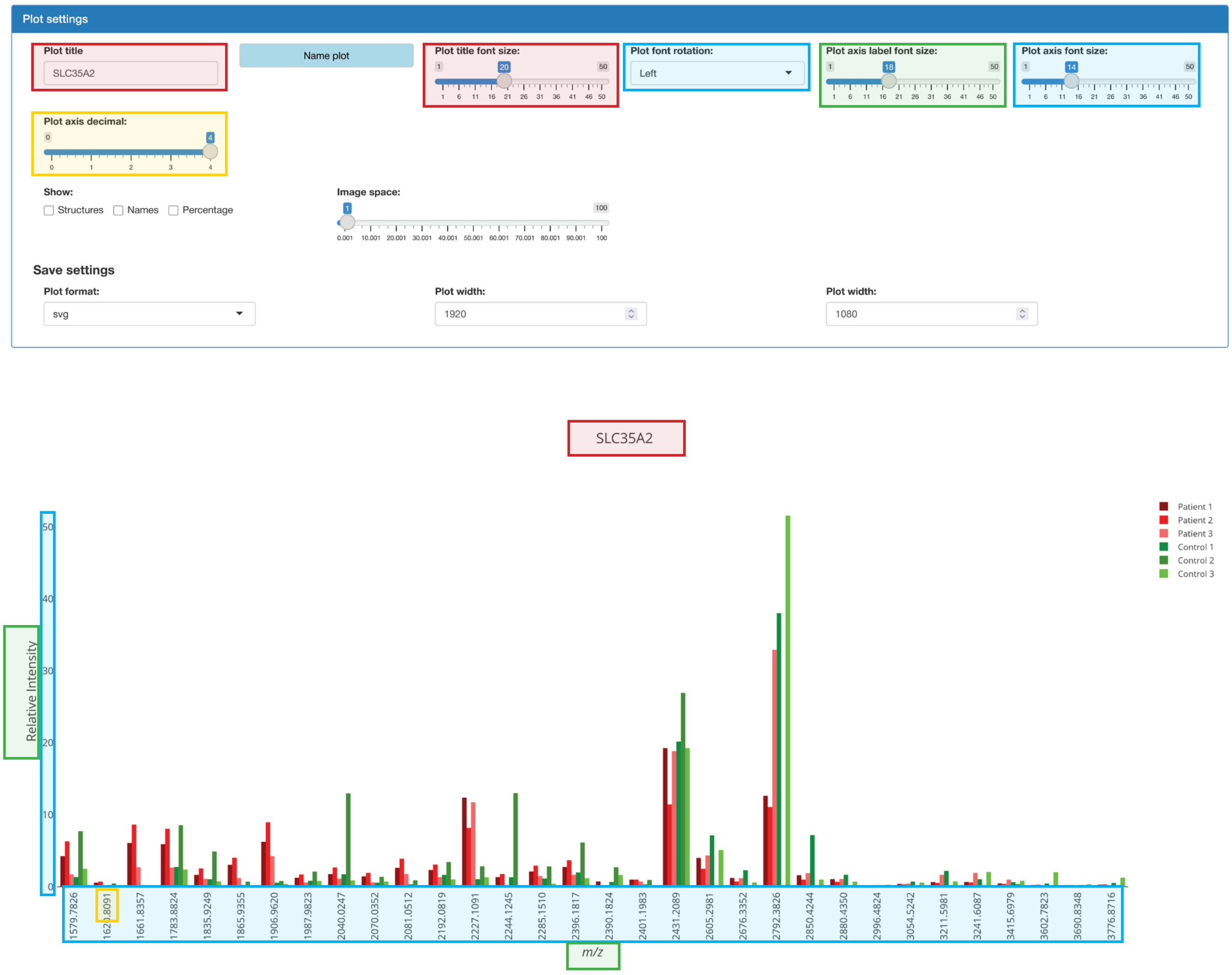

Figure 22. Sample-plot settings: (A) title and axis controls; (B) resulting changes in the plot.

3.6.3 Information displayed above the bars

The Show control determines which types of supplementary information are displayed above the bar groups (Figure 23). Any combination of the following options can be selected:

- Structures: Displays the structure image associated with the value from the selected reference Images column. A structure is displayed only if a corresponding image is available in the configured image directory. If no image is available for an entry, no structure is added above that category. When Structures is selected, the Image size control appears. Use it to increase or decrease the displayed structures.
- Names: Displays the value from the selected reference Names column above each *m/z* category. When Names is selected, two additional controls appear: Names size changes the text size, and Names angle controls the text orientation
- Percentage: In relative-intensity mode, this displays the value of each bar as a percentage, rounded to one decimal place. When Percentage is selected, Percentage size controls the size of these labels.
- Image space controls the vertical distance between the bars and supplementary annotations. Increase it when percentages, names, or structures overlap the bars or one another. Reduce it when the annotations appear too far above the data.

The Info checklist can be used in combination with these settings. The Show options select the types of information used throughout the plot, whereas the Info checklist determines the individual *m/z* categories for which that information is displayed.

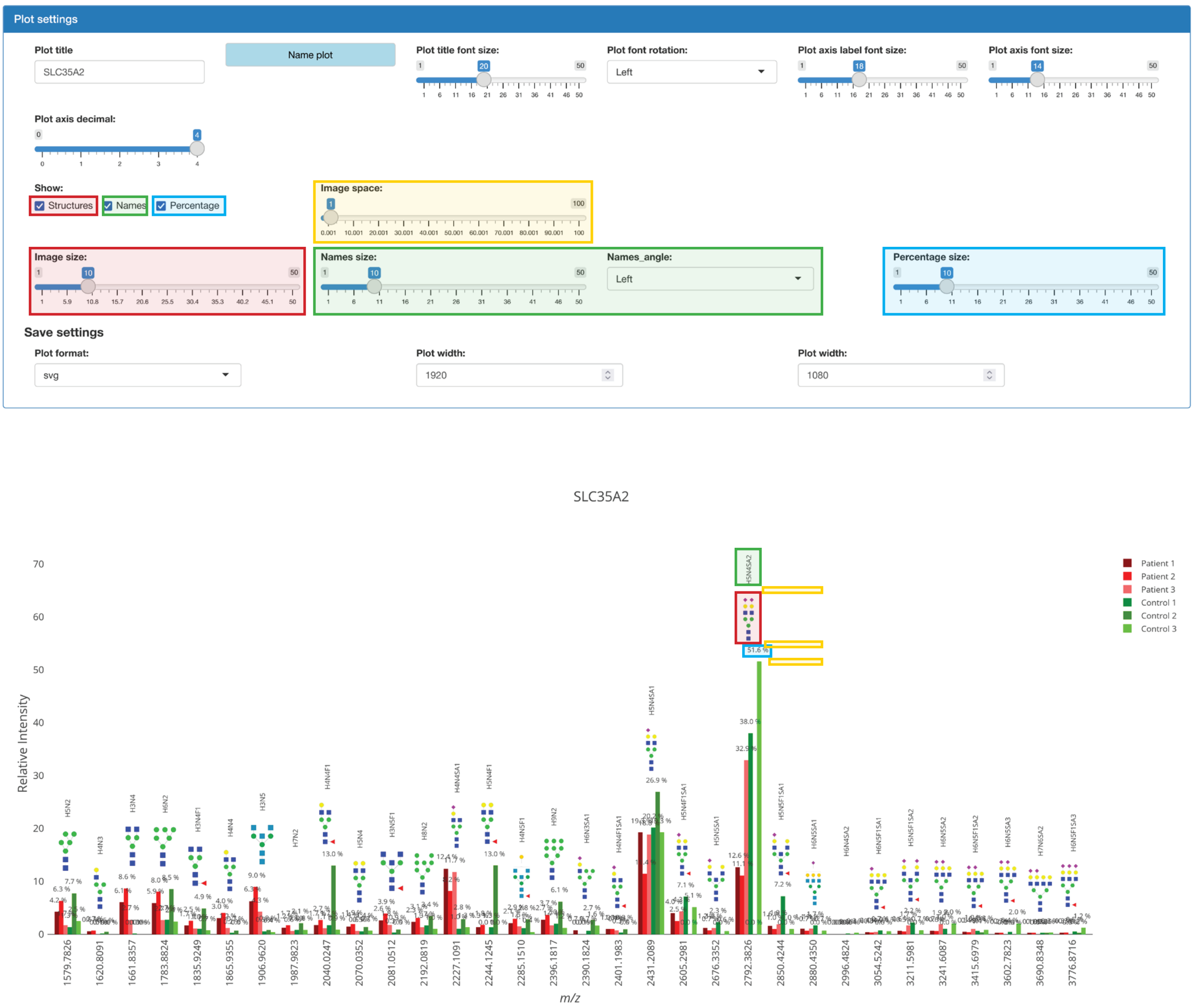

Figure 23. Controls for displaying structures, reference names, and values above sample bars.

Zooming the plot

To zoom in on a region of the plot, drag a rectangle with the left mouse button (Figure 24). To exit the zoomed area/reset the view, doube-click on an empty part of the plot.

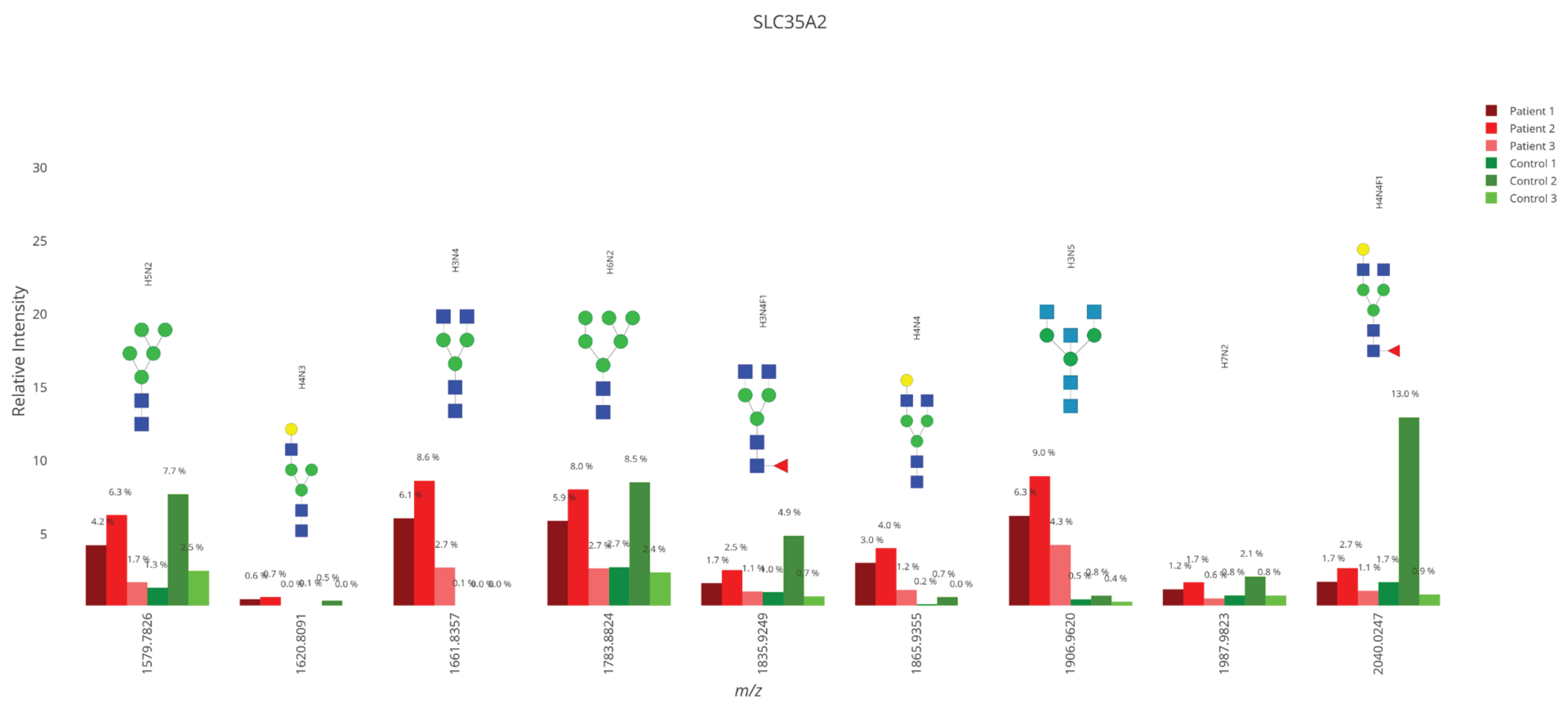

Figure 24. Zoomed view on a part of the plot.

Showing or hiding information above individual bar groups

The Info checklist controls the supplementary information displayed above each *m/z* category. It does not control the bars themselves. Each entry corresponds to one of the available reference *m/z* values. When an entry is unchecked (Figure 25), all supplementary annotations for that reference entry are hidden, including any selected:

– Structure image
– Reference name
– Displayed intensity value

The bars remain visible unless they are removed by the *m/z* filter or hidden by the minimum-value plot filter. When an *m/z* value is removed from the main *m/z* checklist, its corresponding Info checkbox is automatically cleared and disabled because there is no longer a bar category to annotate. The second Select/Deselect all button controls the information checkboxes for the currently active *m/z* values. It does not restore annotations for reference entries that have been removed from the main analysis. The same Info selections are applied to the sample plot and the group plot.

3.6.4 Saving the sample plot

The Save settings section configures image export through the camera icon in the Plotly toolbar.

Plot format: The following formats are available: PNG; SVG; JPEG; WebP. SVG is suitable for vector editing and publication layouts. The raster formats are useful when a fixed-pixel image is required.

Plot width and **Plot height:** Sets the exported image dimensions in pixels. The defaults are: width: 1920; height: 1080. These settings affect the downloaded image and do not change the dimensions of the plot displayed in the browser.

To export the plot move the cursor over the plot to display the Plotly toolbar and click the camera icon (Figure 26).

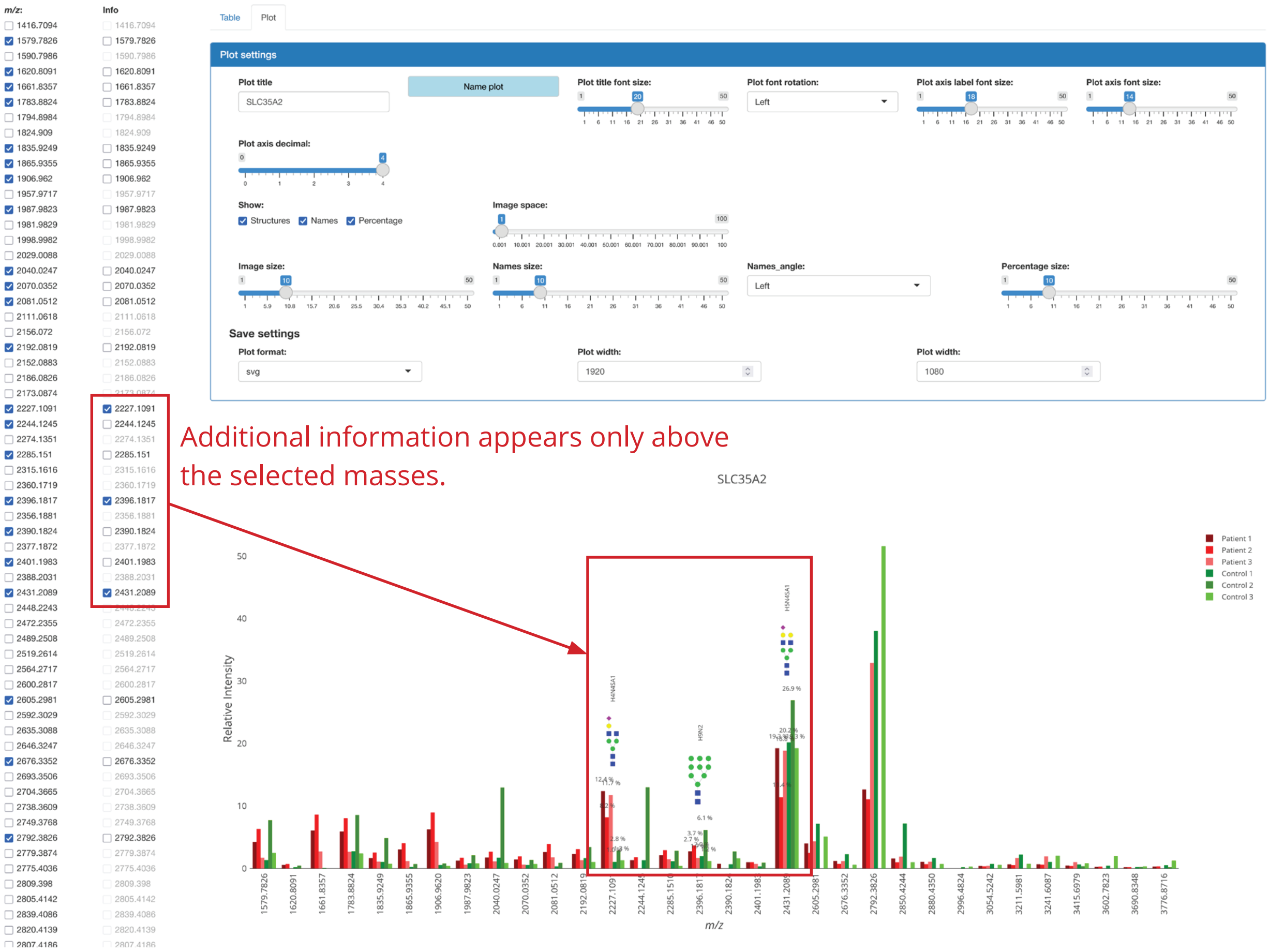

Figure 25. The Info checklist. Deselecting an entry hides the annotations above that m/z category while retaining its bars.

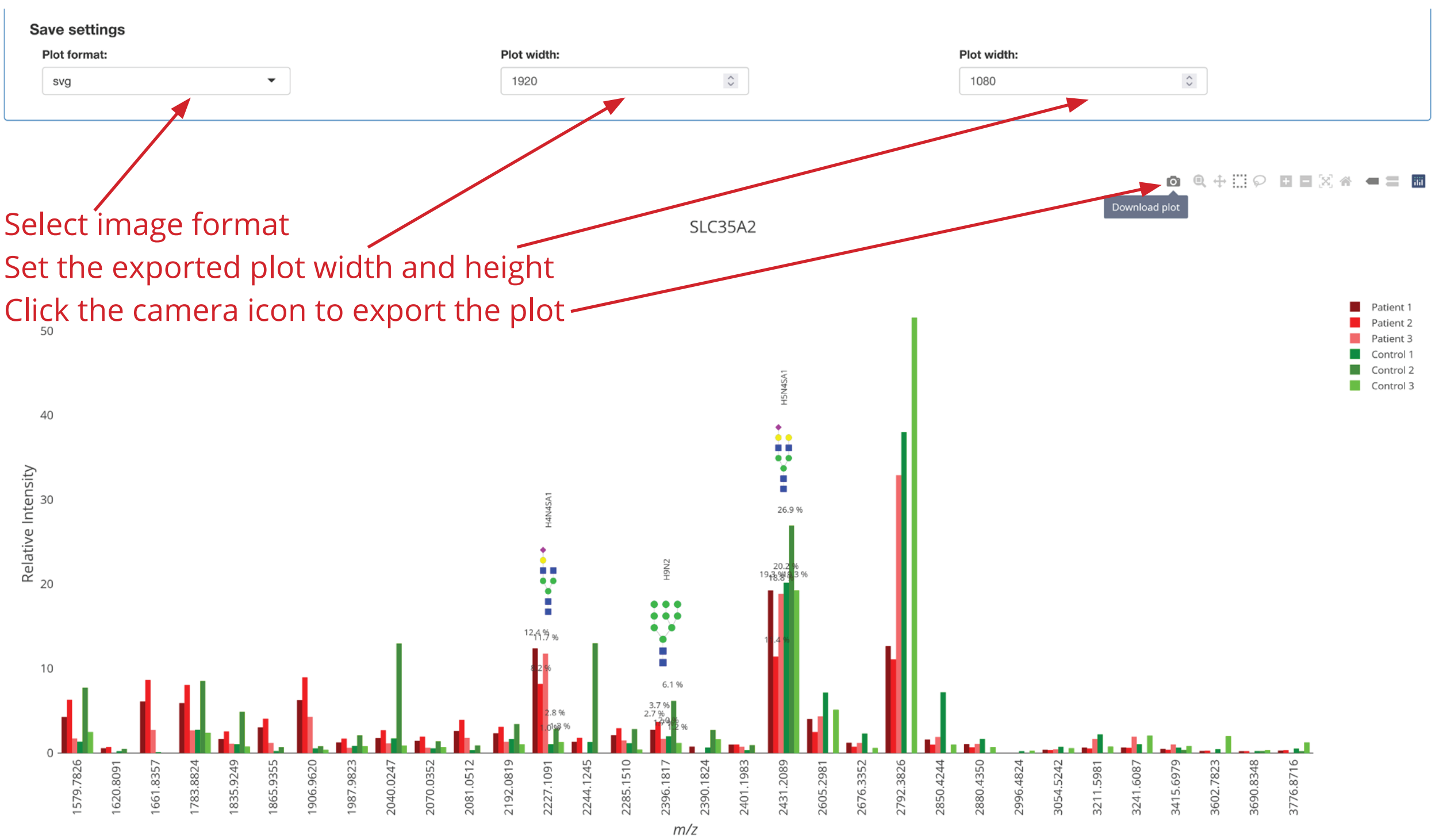

Figure 26. The plot export settings and button.

4 Group analysis

The Groups tab summarizes the selected samples into two or more user-defined groups and provides group-level descriptive statistics, plots, and statistical comparisons.

Since most controls behave in the same way as their equivalents in the Samples tab, the following features are not described again in this chapter:

- renaming groups and selecting group colors;
- selecting or deselecting reference *m/z* values;
- recalculating relative intensities;
- applying the minimum-value plot filter;
- showing or hiding structures, names, and values above bars;
- changing the plot title, fonts, label rotation, and decimal places;
- exporting tables and plots.

For these controls, follow the corresponding instructions in Chapter 3: Sample analysis. The main differences are that the group table reports summary statistics, each bar in the group plot represents a group mean, and the group plot can display SD or SEM error bars.

4.1 Creating groups and assigning samples

At least two samples must be selected before group analysis can be performed (Figure 27).

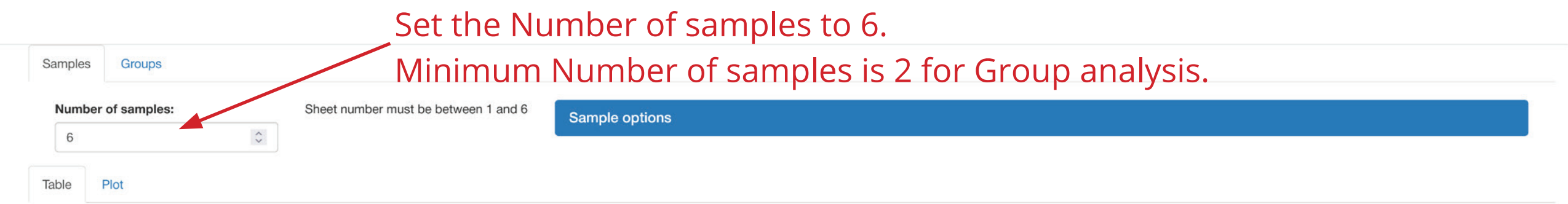

Figure 27. Number of samples in the Samples tab.

Open the Groups tab and enter the required value in Number of Groups (Figure 26.). The minimum number of groups is two. *massMatchR* creates one sample-selection checklist for each group. Every checklist contains all samples currently included in the analysis.

To create the groups:

1. Enter the required number in Number of Groups.
2. Select the samples belonging to each group.
3. Make sure that every group contains at least one sample.
4. Rename the groups and assign their colors, if required.

For example, when six samples consist of three patient samples and three controls, create two groups and assign the samples as follows:

- Group1: SLC35A2-CDG 1, SLC35A2-CDG 2, SLC35A2-CDG 3 (or Patient 1, Patient 2 and Patient 3, if you followed along the user manual and renamed the samples as described in chapter 3.2. Rename samples).
- Group2: Control 1, Control 2, Control 3.

The group table, group plot, and statistical results are generated only after at least one sample has been assigned to every group.

Important Note

- The application allows the same sample to be selected in more than one group. It also allows samples to remain unassigned. However, the available statistical procedures are intended for independent groups.

For a standard independent-group analysis:

- Assign each sample to only one group.
- Do not duplicate samples across groups.
- Ensure that all samples required for the analysis are assigned.
- Confirm the assignments after changing the number or names of samples.

Changing the number of samples, sample names, or number of groups regenerates and resets the group-assignment controls. Review the group assignments after making any of these changes.

Group names and colors are configured in the Group options panel. These controls behave in the same way as the sample-name and sample-color controls described in Chapters 3.2 and 3.3 Group names are entered in group order and separated by semicolons (Figure 29).

4.2 Group results table

The Group Table tab summarizes the selected sample values separately for every group and reference *m/z* value.

For each group, *massMatchR* calculates:

- Arithmetic mean
- Standard deviation (SD)
- Standard error of the mean (SEM)

The reference Names and *m/z* columns are followed by the three columns described above for every group (Figure 29).

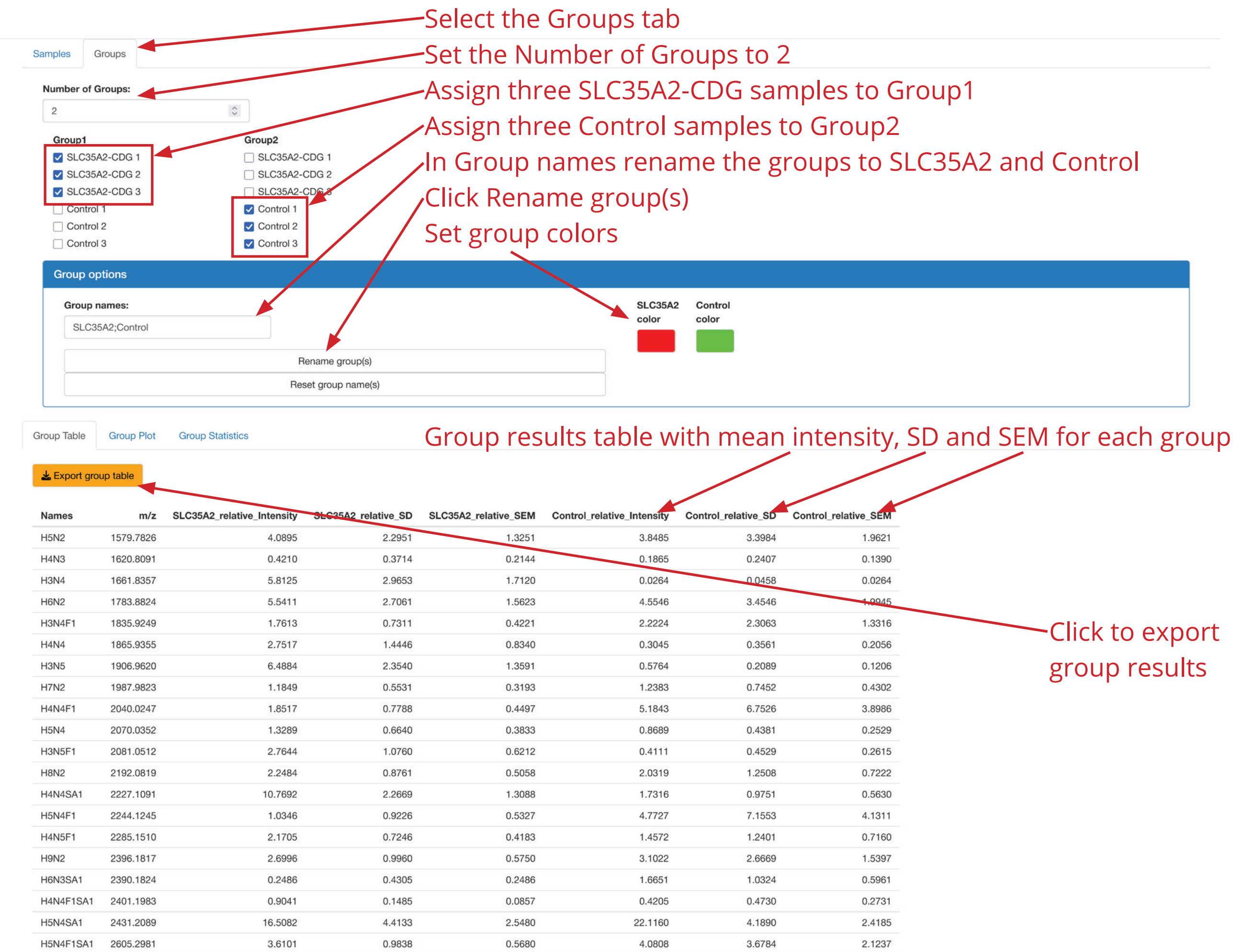

Names	m/z	SLC35A2_relative_Intensity	SLC35A2_relative_SD	SLC35A2_relative_SEM	Control_relative_Intensity	Control_relative_SD	Control_relative_SEM
H5N2	1579.7826	4.0895	2.2951	1.3251	3.8485	3.3984	1.9621
H4N3	1620.8091	0.4210	0.3714	0.2144	0.1865	0.2407	0.1390
H3N4	1661.8357	5.8125	2.9653	1.7120	0.0264	0.0458	0.0264
H6N2	1783.8824	5.5411	2.7061	1.5623	4.5546	3.4546	1.9945
H3N4F1	1835.9249	1.7613	0.7311	0.4221	2.2224	2.3063	1.3316
H4N4	1865.9355	2.7517	1.4446	0.8340	0.3045	0.3561	0.2056
H3N5	1906.9620	6.4884	2.3540	1.3591	0.5764	0.2089	0.1206
H7N2	1987.9823	1.1849	0.5531	0.3193	1.2383	0.7452	0.4302
H4N4F1	2040.0247	1.8517	0.7788	0.4497	5.1843	6.7526	3.8986
H5N4	2070.0352	1.3289	0.6640	0.3833	0.8689	0.4381	0.2529
H3N5F1	2081.0512	2.7644	1.0760	0.6212	0.4111	0.4529	0.2615
H8N2	2192.0819	2.2484	0.8761	0.5058	2.0319	1.2508	0.7222
H4N4SA1	2227.1091	10.7692	2.2669	1.3088	1.7316	0.9751	0.5630
H5N4F1	2244.1245	1.0346	0.9226	0.5327	4.7727	7.1553	4.1311
H4N5F1	2285.1510	2.1705	0.7246	0.4183	1.4572	1.2401	0.7160
H9N2	2396.1817	2.6996	0.9960	0.5750	3.1022	2.6669	1.5397
H6N3SA1	2390.1824	0.2486	0.4305	0.2486	1.6651	1.0324	0.5961
H4N4F1SA1	2401.1983	0.9041	0.1485	0.0857	0.4205	0.4730	0.2731
H5N4SA1	2431.2089	16.5082	4.4133	2.5480	22.1160	4.1890	2.4185
H5N4F1SA1	2605.2981	3.6101	0.9838	0.5680	4.0808	3.6784	2.1237

Figure 29. Group setup and results: assigning samples, renaming groups, selecting colors, viewing mean/SD/SEM values, and exporting the group table.

4.2.1 Mean

For each reference *m/z* value, the group mean is calculated from the intensities of all samples assigned to that group:

$$\bar{x} = \frac{1}{n}\sum_{i=1}^{n} x_i$$

where x_i is the intensity in an individual sample and n is the number of samples assigned to the group.

The group mean is calculated from either:

- relative intensities, when Relative mode is selected; or
- measured intensities, when Absolute mode is selected.

When relative intensities are used, the values included in the group mean also depend on the Recalculate setting described in Chapter 3.5.2.

4.2.2 Standard deviation

The standard deviation describes the variation among the sample values assigned to the group:

A larger SD indicates greater variability among the samples in the group.

$$SD = \sqrt{\frac{\sum_{i=1}^{n}(x_i - \bar{x})^2}{n-1}}$$

4.2.3 Standard error of the mean

The standard error of the mean is calculated as:

SEM describes the uncertainty of the estimated group mean. It generally decreases as the number of samples increases.

$$SEM = \frac{SD}{\sqrt{n}}$$

SD vs SEM

SD and SEM represent different quantities and should not be interpreted interchangeably:

- SD describes variation among the observations.
- SEM describes the precision of the estimated mean.

Groups containing one sample

Sample standard deviation cannot be estimated from a single observation. In the current implementation, when a group contains only one sample, the undefined SD and SEM values are replaced with 0. This value should not be interpreted as evidence that the group has no biological or technical variability. It indicates only that variability cannot be estimated from one sample. Use at least two samples per group when SD or SEM is required, and use an appropriate number of independent replicates for statistical analysis.

4.2.4 Filters applied to the group table

The group table follows the same analysis settings as the sample table, including:

- the selected samples;
- group assignments;
- selected reference *m/z* values;

- relative or absolute intensity mode;
- the Recalculate setting.

The minimum-value filter and the Info checklist affect only the plot and do not remove values from the group table.

4.2.5 Exporting the group results table

Click Export group table button (Figure 29) to download the displayed group summaries as an Excel workbook. The table-export procedure is the same as that described for the sample table.

4.3 Group plot and error bars

Open the Group Plot tab to visualize the group means. The main difference from the sample plot is the meaning of each bar:

- in the sample plot, each bar represents one sample;
- in the group plot, each bar represents the mean of the samples assigned to one group.

The colors are taken from the Group options panel, and the legend displays the current group names. All general plot settings behave as described for the sample plot, including:

- title and title size;
- axis-label and tick-label sizes;
- *m/z* label rotation;
- displayed decimal places;
- structure images;
- reference names;
- values above bars;
- image spacing and annotation sizes;
- plot format and image dimensions.

The group plot adds controls for SD and SEM error bars.

4.3.1 Selecting error bars

Use the SEM/SD control (Figure 30) to choose the variability measure displayed above and below each group mean:

- none: Displays group means without error bars; select none when error bars are not required.
- SEM: Displays the mean ± SEM; use SEM to show the precision of the estimated group mean.
- SD: Displays the mean ± SD. Use SD to show variation among the samples.

The error bars are calculated separately for every group and reference *m/z* value from the same sample values used in the group table. The selected error bars are descriptive only. Changing them does not alter group means, tables, or statistical-test results.

4.3.2 Error-bar appearance

Two additional controls modify the appearance of the error bars (Figure 30):

- Error bar thickness: Changes the thickness of the vertical error-bar lines.
- Error bar width: Changes the width of the horizontal caps.

These settings affect only the visual appearance of the group plot. When structures, names, or values are displayed above the bars, *massMatchR* adjusts their vertical position to provide space for the selected SD or SEM error bars.

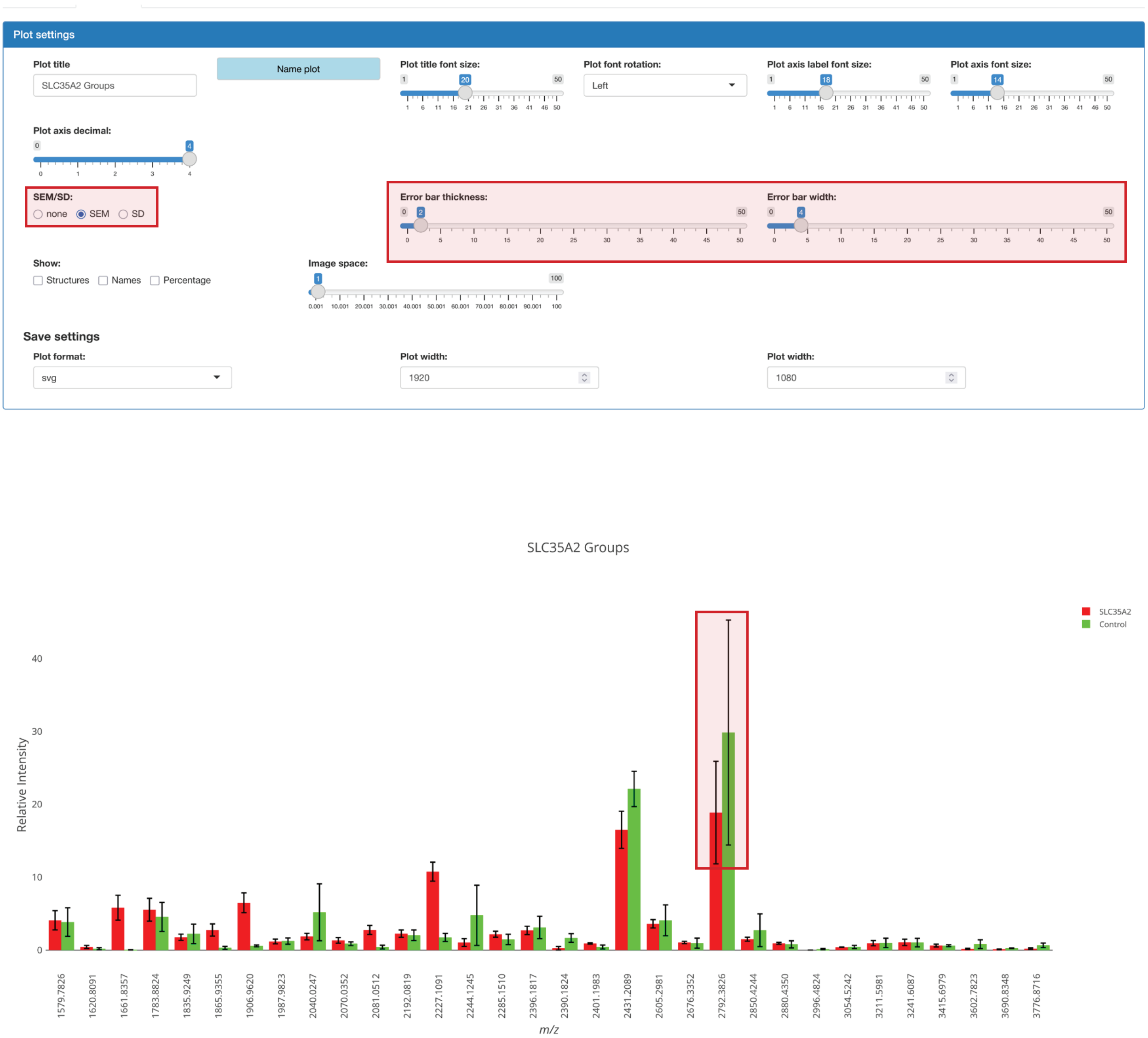

Figure 30. Group plot with the SD/SEM selector and error-bar appearance controls.

4.3.3 Minimum-value filtering

In the group plot, Filter by min. value is applied to the calculated group means rather than to individual sample values. A group bar is hidden when its mean is below the selected threshold. If all group means for a reference *m/z* value are hidden or equal to zero, that complete *m/z* category is removed from the plot. This remains a visualization filter. It does not remove the corresponding values from the group table or statistical analysis.

4.4 Group statistical analysis

The Group Statistics tab performs a separate statistical analysis for every selected reference *m/z* value. The tests use the individual sample intensities assigned to the groups. They do not perform statistical testing on the already calculated group means.

The available controls include the distribution assumption, primary statistical test, p-value adjustment method, post-hoc test, and significance thresholds.

4.4.1 Distribution assumption

Use Distribution assumption to select the family of statistical tests:

- Normal (parametric): Select this option when the data and experimental design support the assumptions of parametric testing.
- Non-normal / unknown: Select this option when a normal-distribution assumption is not appropriate or cannot be justified.

massMatchR does not automatically evaluate:

- normality;
- equality of variances;
- independence of observations;
- sample size adequacy;
- suitability of the selected statistical test.

These conditions must be evaluated by the user according to the experimental design and properties of the data.

4.4.2 Analysis of two groups

When exactly two groups are defined, the available primary tests depend on the selected distribution assumption.

Parametric tests

When Normal (parametric) is selected, choose one of the following tests:

- Independent t-test: The independent t-test compares the means of two independent groups and assumes equal variances. Select this test only when the equal-variance assumption is appropriate.
- Welch t-test: The Welch t-test compares the means of two independent groups without assuming equal variances. It is generally more appropriate when group variances or sample sizes differ.

Non-parametric test

When Non-normal / Unknown distribution is selected, the user interface changes the available statistical tests the following non-parametric test:

- Mann-Whitney U test: The Mann-Whitney U test, also known as the Wilcoxon rank-sum test, compares two independent groups without requiring a normal-distribution assumption. It tests differences in the distributions or ranks of the observations and should not automatically be interpreted as a test of arithmetic means.

4.4.3 Analysis of more than two groups

When more than two groups are defined, the user interface displays different parametric and non-parametric tests depending on the distribution.

Parametric test

When Normal distribution is selected, *massMatchR* can perform:

- One-way ANOVA: It evaluates whether evidence exists that at least one group mean differs from

the others. A significant ANOVA result does not identify which groups differ. Pairwise differences are evaluated with the selected post-hoc test.

Non-parametric test

When distribution is non-normal / unknown, the available test is:

- Kruskal-Wallis test: It evaluates whether the distributions or ranks differ among the groups. A significant Kruskal-Wallis result does not identify the differing group pairs. Pairwise differences are evaluated with Dunn's post-hoc test.

4.4.4 Post-hoc tests

Post-hoc controls appear only when more than two groups are analyzed.

Post-hoc tests after one-way ANOVA

The following options are available:

- Tukey HSD: Tukey's honestly significant difference test performs pairwise comparisons while controlling for multiple comparisons. The reported Tukey p-values are already adjusted.
- Fisher LSD (no correction): Fisher's least significant difference procedure performs pairwise t-tests without multiple-testing correction. Because no correction is applied, the probability of false-positive results increases as the number of comparisons increases.
- Pairwise t-test (Bonferroni): This option performs pairwise t-tests and adjusts the pairwise p-values using the Bonferroni method.
- Pairwise t-test (BH/FDR): This option performs pairwise t-tests and adjusts the pairwise p-values using the Benjamini-Hochberg method.

Post-hoc test after Kruskal-Wallis

When the Kruskal-Wallis test is selected, *massMatchR* performs Dunn's test for pairwise comparisons. The correction applied to the Dunn-test p-values follows the selected P-value adjustment method.

4.4.5 P-value adjustment

The P-value adjustment method controls correction of the primary-test p-values across all tested reference *m/z* values. The available methods are:

- None: No correction is applied.
- Bonferroni: Controls the family-wise error rate using Bonferroni correction.
- BH: Applies the Benjamini-Hochberg false-discovery-rate procedure.

The selected adjustment also controls Dunn-test correction following a Kruskal-Wallis analysis. Pairwise tests following ANOVA use the correction specified by the selected post-hoc method.

4.4.6 Significance thresholds

Three user-defined thresholds determine which pairwise results are marked as significant.

1. P-value Threshold: A result passes this criterion when p < selected raw p-value threshold. The default raw p-value threshold is 0.05.
2. Adjusted P-value Threshold: A result passes this criterion when padjusted < selected adjusted p-value threshold. The default adjusted p-value threshold is 0.05.
3. Absolute Log2FoldChange Threshold: A result passes the effect-size criterion when |log2FC| ≥ selected log2 fold-change threshold. The default absolute log2 fold-change threshold is 1.

A pairwise comparison is marked significant only when it satisfies both the relevant p-value threshold and the absolute log2 fold-change threshold:

- For raw p-values: p < pthreshold and |log2FC| ≥ log2FCthreshold
- For adjusted p-values: padjusted < padjusted threshold and |log2 FC| ≥ log2FCthreshold

For analyses with more than two groups, the log2 fold-change criterion is applied to the individual post-hoc comparisons, not to the overall ANOVA or Kruskal-Wallis test.

4.4.7 Interpreting the statistical-results table

Two-group analysis

For two groups, the results include:

- the reference name;
- the reference *m/z* value;
- the log2 fold-change between the two group means;
- the primary-test p-value;
- a raw significance indicator;
- the adjusted p-value;
- an adjusted significance indicator.

Analysis of more than two groups

For more than two groups, the results include:

- the overall ANOVA or Kruskal-Wallis p-value;
- the multiple-testing-adjusted omnibus p-value;
- pairwise post-hoc log2 fold-changes;
- pairwise raw p-values;
- pairwise raw significance indicators;
- pairwise adjusted p-values;
- pairwise adjusted significance indicators.

The omnibus test (One-way ANOVA or Kruskal-Wallis test) indicates whether evidence of a difference exists somewhere among the groups. The post-hoc results identify the specific group comparisons associated with those differences.

4.4.8 Exporting the statistical-results table

Click Export group stats to download the complete statistical-results table as an Excel workbook.

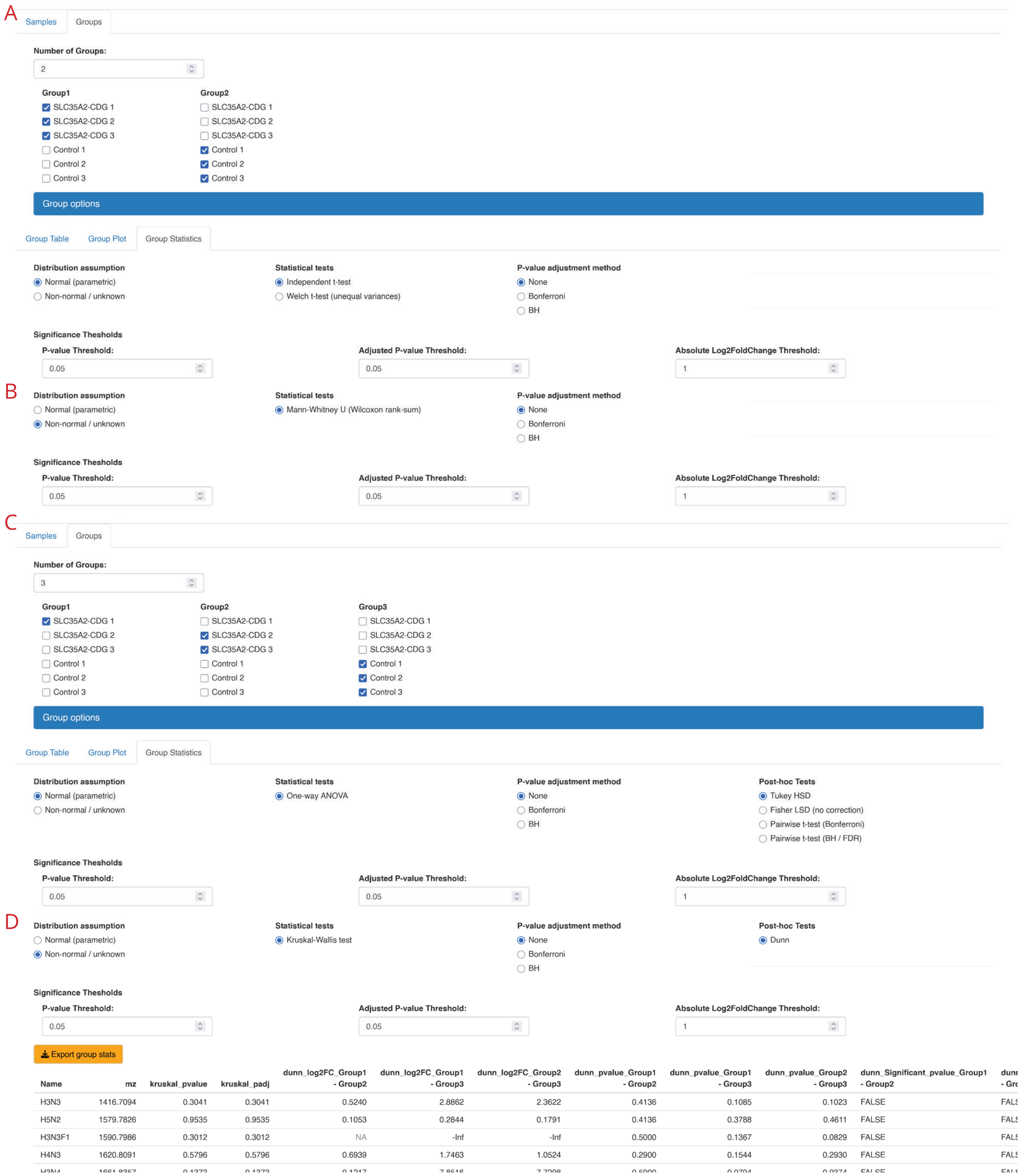

Figure 31. Statistical-analysis options in the Group Statistics tab: (A) independent t-test and Welch t-test for two groups with a normal-distribution assumption; (B) Mann–Whitney U test for two groups with a non-normal or unknown distribution; (C) one-way ANOVA and the available post-hoc tests for three groups with a normal-distribution assumption; (D) Kruskal–Wallis test and Dunn's post-hoc test for three groups with a non-normal or unknown distribution.
